# Nonlinear latent representations of high-dimensional task-fMRI data: Unveiling cognitive and behavioral insights in heterogeneous spatial maps

**DOI:** 10.1371/journal.pone.0308329

**Published:** 2024-08-08

**Authors:** Mariam Zabihi, Seyed Mostafa Kia, Thomas Wolfers, Stijn de Boer, Charlotte Fraza, Richard Dinga, Alberto Llera Arenas, Danilo Bzdok, Christian F. Beckmann, Andre Marquand

**Affiliations:** 1 Donders Institute for Brain, Cognition and Behavior, Radboud University Nijmegen, Nijmegen, the Netherlands; 2 Department for Cognitive Neuroscience, Radboud University Medical Center Nijmegen, Nijmegen, the Netherlands; 3 MRC Unit for Lifelong Health & Ageing, University College London (UCL), London, United Kingdom; 4 Department of Psychiatry, University Medical Center Utrecht, Utrecht, the Netherlands; 5 NORMENT, KG Jebsen Centre for Psychosis Research, Division of Mental Health and Addiction, Oslo University Hospital & Institute of Clinical Medicine, University of Oslo, Oslo, Norway; 6 Department of Psychiatry and Psychotherapy, Tübingen Center for Mental Health, University of Tübingen, Tübingen, Germany; 7 Multimodal Imaging and Connectome Analysis Lab, McConnell Brain Imaging Centre, Montreal Neurological Institute and Hospital, McGill University, Montreal, Quebec, Canada; 8 Mila ‐ Quebec Artificial Intelligence Institute, Montreal, Quebec, Canada; 9 Centre for Functional MRI of the Brain, University of Oxford, Oxford, United Kingdom; 10 Department of Neuroimaging, Institute of Psychiatry, Psychology, & Neuroscience, King’s College London, London, United Kingdom; Bocconi University: Universita Bocconi, ITALY

## Abstract

Finding an interpretable and compact representation of complex neuroimaging data is extremely useful for understanding brain behavioral mapping and hence for explaining the biological underpinnings of mental disorders. However, hand-crafted representations, as well as linear transformations, may inadequately capture the considerable variability across individuals. Here, we implemented a data-driven approach using a three-dimensional autoencoder on two large-scale datasets. This approach provides a latent representation of high-dimensional task-fMRI data which can account for demographic characteristics whilst also being readily interpretable both in the latent space learned by the autoencoder and in the original voxel space. This was achieved by addressing a joint optimization problem that simultaneously reconstructs the data and predicts clinical or demographic variables. We then applied normative modeling to the latent variables to define summary statistics (‘latent indices’) and establish a multivariate mapping to non-imaging measures. Our model, trained with multi-task fMRI data from the Human Connectome Project (HCP) and UK biobank task-fMRI data, demonstrated high performance in age and sex predictions and successfully captured complex behavioral characteristics while preserving individual variability through a latent representation. Our model also performed competitively with respect to various baseline models including several variants of principal components analysis, independent components analysis and classical regions of interest, both in terms of reconstruction accuracy and strength of association with behavioral variables.

## Background

An important challenge in the application of machine learning to neuroimaging is to find an optimal low dimensional summary or representation of the complex spatial information encoded in brain images into a biologically interpretable readout that preserves important relationships between data points. These representations can be used to understand inter-individual differences, ascertain association between neuroimaging data and cognitive variables, and identify biomarkers that can be used to understand and make predictions on the basis of the biological underpinnings of both healthy and disordered mental states [1–5].

Deep neural network models are one candidate for providing such a representation. However neuroimaging studies have traditionally had a limited number of high-dimensional datasets, which until recently had hindered the use of complex deep neural network models for a time due to the curse of dimensionality [6]. The recent increase in the availability of large-scale neuroimaging data has provided a great opportunity to move toward using complex nonlinear methods, for example, based on deep learning approaches [7–14]. Many deep learning studies in neuroimaging use hand-crafted features [5, 15–18] e.g., regions of interest (ROIs) or image-derived phenotypes (IDPs)- which are potentially suboptimal for prediction because: first, hand-crafted features may fail to capture complex structural or functional characteristics of the brain. Individual differences are often encoded in intricate and overlapping patterns in the brain that are important for understanding its relationship with behavior. Hand-crafted features might not represent these complexities accurately, leading to inferior predictions. Second, these studies do not benefit from the strength of deep neural networks in automatically learning the optimal representation from the data, achieved when the model converges, such as through the use of convolutional filters. This highlights the need for more effective methods to learn representations of neuroimaging data that can accurately predict clinical and cognitive variables. Particularly in task fMRI studies, which are designed to explore mappings from brain activations to cognition and behavior, numerous challenges exist. These includes the extensive heterogeneity across individuals, finding a comprehensive representation, and the need for a reliable reference to compare the activations [19–25]. Consequently, using hand-crafted features potentially leads to losing relevant information, such as inter-individual differences in functional anatomy [5, 26]. In these scenarios, learning a representation of high-dimensional neuroimaging data—rather than using predefined ROIs, for example—may enable a better understanding of individual variations and lead to more accurate predictions of clinical and cognitive measures. Such latent representations allow us to reduce the data dimensionality and extract only the essential features. In other words, a latent representation maps complex and high-dimensional data into a reduced, low-dimensional space [27]. Thus, our research question is: How can we leverage nonlinear techniques to learn a generic or general-purpose latent representation of task-fMRI (tfMRI) data that is not bound to a specific task and accurately predicts a wide range of cognitive and clinical variables?

Motivated by the limitations of existing approaches and the potential benefits of a general-purpose latent representation, we propose a method to learn such a representation from tfMRI data. Most applications of deep learning in neuroscience focus on learning a latent representation optimized for a single supervised learning problem, such as predicting age or sex (e.g., [7, 11, 28, 29]). However, this approach may reduce the generalizability of the learned latent representation to other problems. Our approach aims to overcome this limitation by learning a general-purpose latent space that is not bound to a specific task but instead captures features from the data that are predictive of wide range of cognitive scores. Numerous efforts have been made towards this end [30–37]. Most of these studies evaluate the data representations on the basis of specific measures like reconstruction error but this does not necessarily suggest that the latent space captures relevant features. Although linear data-driven transformations like Principal Component Analysis (PCA) and Independent Component Analysis (ICA) [38–42] are widely used for feature representation and dimensionality reduction in neuroimaging, these methods often fail to extract complex nonlinear relationships in the data [43, 44] To address this challenge, we introduce a 3-dimensional semi-supervised autoencoder (AE) that learns a nonlinear latent space representation of tfMRI images, capturing relevant features and their associations with nIDPs.

In this paper, we propose exploring the value of learning a general purpose nonlinear latent space representation of tfMRI contrast images using a 3-dimensional semi-supervised AE.

Autoencoder neural networks are powerful tools in various applications in neuroimaging studies, from image segmentation to abnormality detection and latent representation [8, 9, 33, 45–48]. Briefly, an autoencoder is a deep neural network architecture consisting of two parts: an encoder and a decoder. The encoder projects the inputs to a lower-dimensional latent space using a non-linear transformation. The decoder translates back the latent space to the original space by reconstructing the inputs [49]. Complementary to existing approaches, we demonstrate how we can control the learned latent representation by incorporating a supervised learning term into the reconstruction, that is, within a joint optimization framework. In our approach, we tailor the search space by adding age and sex to the loss function minimized by the model, ensuring the learned latent representation is not limited to a specific task. Our approach, in contrast to many previous methods, does not require the prior specification of regions of interest (i.e. which can be used as nodes in the network). Instead, it can learn overlapping representations, use the full range of spatial patterns in the fMRI signal, and leverage the strengths of deep learning, such as learning convolutional filters that capture low-level features of the images.

Having learned the latent representation, we then assess whether it demonstrates a stronger association with cognitive, clinical and demographic variables ‐ collectively referred to as ‘non-imaging-derived phenotypes’ (nIDPs) ‐ compared to data in the original space (e.g., mapping from raw image data or hand-crafted features to behavioral scores) or to traditional linear dimensionality reduction techniques such as PCA.

**Fig 1:**
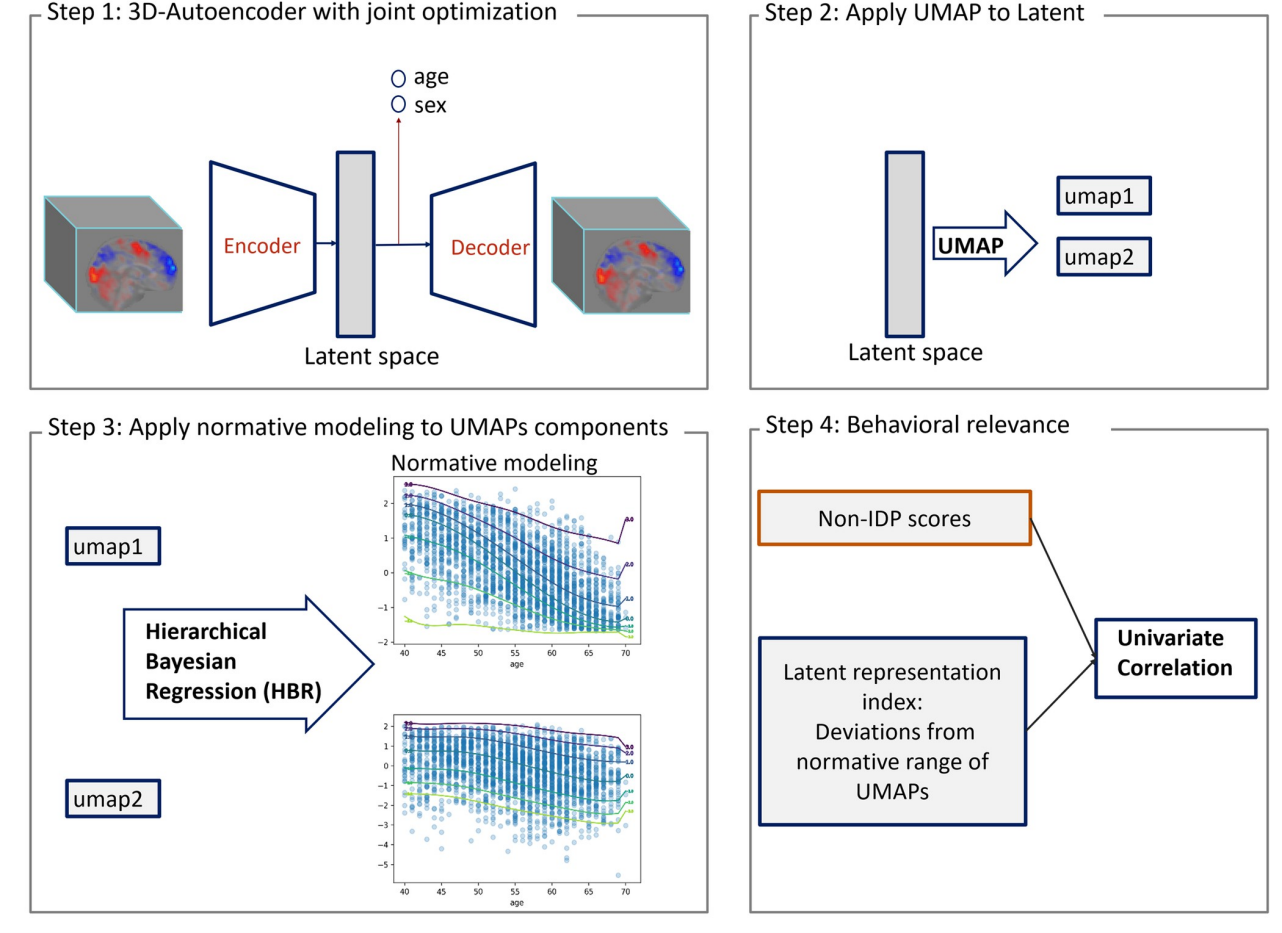
Method overview: step1) training semi-supervised AE model with joint optimization of age and sex prediction. Step2) applying UMAP transformation to the latent variables of semi-supervised AE. step3) applying HBR normative modeling to the components of UMAPs. Step4) measuring the correlation of non-imaging scores (behavioral, cognitive and clinical scores ) and the deviation value from normative range of UMAP components (latent representation index).

More specifically, in a fully data-driven approach shown in Fig 1, we showed that there is useful information about the data in the nonlinear latent space that is not fully captured by a linear data representation and that such information can be extracted using a hierarchical non-linear autoencoder architecture with joint optimization for age and sex prediction [5, 8]. Here, we employed an autoencoder with an architecture designed from the ground up for tfMRI data and provided a method for visualizing, exploring, and interpreting the learned representation. Last, in this study, we aimed to illustrate how our model can be employed to understand inter-individual differences in brain activity. To achieve this, we applied a normative model [50–52] to a compressed representation of the latent variables, which was derived using the Uniform Manifold Approximation and Projection (UMAP) technique. This approach allowed us to separate age-related variation from other sources of variability, thereby providing a more detailed insight into the factors influencing brain function. We use these deviations for detecting associations with nIDPs. We first trained our model with multi-task fMRI data derived from the Human Connectome Project (HCP) [19] which provides whole-brain coverage across a range of cognitive tasks. We then fine-tuned the network using UK Biobank dataset [23]. Our experimental results show that our nonlinear data representation provides a strong foundation for subsequent analysis of brain-behavior mappings and results in strong associations between our latent index and unseen nIDPs.

## Methods

### Data

Two different datasets were used in this study. This first dataset consists of tfMRI data from the HCP [19] S500 release. The second tfMRI dataset is from the 2020 UK Biobank imaging release [53].

#### HCP

We used tfMRI contrast data from 468 participants in total (187 males and 281 females, Age = 29.2±3.5) from seven different behavioral and cognitive tasks (emotion processing, gambling, language, relational processing, social cognition, motor, working memory) across 86 contrasts (86 sub-tasks). These tasks served as the basis in previous brain-imaging work [54, 55]. This yields a total of N≈40K tfMRI scans. The HCP dataset is well suited for this purpose because the task battery covers a wide range of cognitive domains and the neuronal activations associated with the task provide good coverage of the entire brain [20]. The number of participants may vary from task to task; not all the participants have data in all the tasks. While HCP has a large number of samples, the number of participants is relatively small. Therefore, we split data into 5 subsets in a 5-fold cross-validation scheme. The splits are made at the subject level, ensuring that each fold contains all the contrasts for a specified set of subjects. This prevents overly optimistic estimates of generalizability due to the correlations between different contrasts from the same subject. More specifically, in each fold, about 95 participants (20% of the data) were reserved for the test set (N = 8K brain scans) and the rest for the training (N = 32K brain scans, 373 participants). For each fold, we trained a separate model. Moreover, to further guard against overfitting, an independent set of subjects were used to determine the model architecture and optimize hyperparameters (see below and in the S1 File for details).

#### UK Biobank

We used UK Biobank tfMRI contrast data from 20,781 participants and 5 contrasts, in total N≈104K scans (9,860 males, 10,921 females, Age = 54.6 ±7.4). The tfMRI data derived from UK Biobank uses the same paradigm as the emotion task from the HCP with only minor modifications (e.g. to accommodate shorter run length) [23, 53]. We randomly selected N = 15585 of participants for the train set and 5196 for the test set. All the contrast-models employ the same dataset configuration (the test and train sets).

#### Non-imaging-derived phenotypes (nIDP) data

The UK Biobank study provides an extensive number of clinical, behavioral, lifestyle and cognitive scores, which we categorized to seven groups e.g., cognitive phenotypes, lifestyle, and mental health (see S1 File for the full list of categories). We only included the measures that their scores are available more than half of participants. Moreover, in line with previous studies [56, 57] the measures that had same value for more than 80% of the participants were excluded from further analysis.

### Image preprocessing

For both datasets we used the volumetrically preprocessed images in standard reference space provided by the respective consortia [58, 59] (for HCP using the ‘minimally processed’ pipeline [58]). Subsequently the scans were downsampled from 2mm to 3mm voxel resolution to reduce the computational burden then cropped tightly to the whole brain such that the dimension of the image decreased to 56×64×56. The model was trained on the whole-brain contrast images.

### Model architecture

We developed a deep 3D-convolutional autoencoder that learns to encode and decode tfMRI images using HCP data. Since many choices need to be made regarding the architecture of the autoencoder, we performed a pilot study on a subset of data that was discarded before fitting the final model. Here, we selected the architecture for the autoencoder using held-out data (N = 30 participants reserved data, 2580 scans). Full details of this procedure are provided in the S1 File. The final architecture was as follows: Each encoder and decoder of the semi-supervised AE had three hidden convolutional layers with 3x3x3 kernel size. The bottleneck of the model is a dense layer containing 100 nodes. Each layer, except the output layer, was followed by ReLU activation function [60] to add non-linearity and sparsity to the network and to reduce the likelihood of vanishing gradient. The output layer was followed by a linear activation function. To increase the robustness of the model and avoid overfitting, we incorporated drop-out [61] (drop-out level = 0.2) in each layer except the output layer. To avoid the risk of a degenerate solution, where the autoencoder simply learns the identity function, we added Gaussian noise [62] (mean = 0, standard deviation = 0.1) to the input layer to randomly corrupt the data (see S1 File for details about the optimization of the architecture of the semi-supervised AE ).

The loss function to train the model contains two parts; an unsupervised and a supervised loss. The unsupervised loss simply is the mean squared error of the reconstruction image of the noisy image and the original image. The supervised loss is incorporated in order to control of latent space of the autoencoder; Here, we added age and sex as the supervised part of calculating the loss function. Without this, we cannot guarantee that the learned representation would contain any information about relevant demographic features. We used age as a continuous variable rather than a one-hot encoded matrix (i.e. which would effectively treat the regression as a classification problem [63]). This enables us to generalize beyond the age range used in the training dataset, which is important for transfer learning because of potential differences between cohorts. So the training loss is defined by:

$$loss=\lambda {\left(x-\hat{x}\right)}^{2}+(1-\lambda )\left(|y_{age}-{\hat{y}}_{age}|+Binary\;crossentropy\left(y_{sex}-{\hat{y}}_{sex}\right)\right)$$

which x is the input image and *y*_*age*_ and *y*_*sex*_ are age and sex. The first term refers to unsupervised loss which is the usual autoencoder loss and the second term refers to supervised loss. To balance the supervised and unsupervised loss in terms of scale, we used coefficient *λ* which specifies the importance of supervised loss e.g., *λ* = 1 means a completely unsupervised autoencoder (i.e. ‘vanilla AE’).

We also trained our model on the held-out calibration dataset with different range of *λ* = 1, 0.995, 0.95, 0.5, 0.05, 0.005 to select the optimum value of *λ* in terms of unsupervised and supervised loss.

### Training the model

The training data were normalized to have zero mean unit variance across each feature. The layers weights were initialized using Xavier initialization [64]. First, the model was trained using HCP data with 1,000 epochs and using Adam [65] optimizer with an adaptive learning rate. The base learning rate was set at 0.001 and with exponential learning rate decay over each epoch reached 0.0003. Last, the mini‐batch gradient descent was conducted with the size of 10 images.

Having trained the model by HCP, the network was trained again using the same hyperparameters with UKB data. Although, this procedure has a similar motivation to fine-tuning, in fact all the weights were re-estimated (i.e. none of the layers were frozen). We consider this to be the most appropriate approach because the age range is very different across these two datasets. Here, the weights of the trained model by HCP were used as initial weights. The base learning rate was reduced to 0.0003 for training with UKB data to limit significant modifications to the pre-trained weights, thereby preserving the valuable features already learned during the initial training phase.

### Visualising the latent space representation using UMAP

To visualize and evaluate our model quantitatively, we visualized the latent space using a UMAP approach [66] with two components. UMAP,a manifold learning technique similar to t-distributed stochastic neighbor embedding (t-SNE) [67], preserves the local structure of high dimensional data in a nonlinear space. UMAP is superior to tSNE since it better preserves the global structure of data, in addition to its local structure. Furthermore, it is more stable under perturbation or resampling of the data.

To visualize the latent space with two UMAP components, a UMAP model was fitted using latent variables derived from the training set. To avoid over-engineering the results, we applied UMAP with the default parameter settings. More specifically, the size of local neighborhood to learn the manifold structure of the data was set to 15 while the minimum distance of each data in the low dimensional representation was 0.1 in Euclidean distance. Later, this model was applied to the predicted latent variables of test images. We leave further optimization of these parameters for future work.

We provide several simple examples to demonstrate how this representation helps us understand the distinctiveness, overlap, and distribution of different stimulus classes at different scales. For example, the derived latent representation facilitates the assessment of functional differentiation, determines whether different tasks have distinct or overlapping distributions, helps decide whether a gradient-based or discrete representation may be more appropriate for the data. Moreover, the semi-supervised autoencoder allows us to probe the degree to which functional differentiation changes as a function of different covariates (e.g. whether different tasks or contrasts are differentially affected by ageing processes).

To illustrate, we begin by showcasing the functional differentiation among all HCP tasks, then focus specifically on the motor task, which has three different experimental conditions, based on whether participants were moving their tongue (T), the left hand (LH), right hand (RH) or left or right foot (LF/RF; see Barch et al 2013 [20] for details on the task paradigm). To achieve this, we first calculate the center of the latent space for each task, representing the average response. We then transfer these centers back to the original space using the decoder to generate the corresponding brain images. This is of interest because the classical notion of a relatively continuous ‘homunculus’ representation of the motor cortex has recently been challenged suggesting a higher degree of functional differentiation than has previously been appreciated [68].

### Associations with nIDPs data

#### Normative modeling of UMAP

To show how our latent space can be used to provide biomarkers that can be then used to predict external behavioural, clinical, or demographic variables–often referred to as nIDPs–we calculated the linear association between clinical and behavioral measures and the deviations of the UMAP-reduced latent space for UK Biobank data. To establish a baseline for comparison, we additionally calculated the associations of nIDPs with UMAPs derived from PCA (number of components = 100), ICA (number of components = 100), and sub-cortical ROIs. However, since the latent variables are related to age and age has a strong association with many cognitive and behavioral scores, we employed normative modeling on the latent space to separate variation that is principally age-related (encoded by the normative model) from inter-individual differences that manifest as deviations from an expected age-related pattern (encoded in the deviations from the normative model). The normative modeling approach has been used extensively to model heterogeneity in various psychiatric disorders [18, 69, 70]. Briefly, this approach provides a statistical estimation of the distribution of brain measures along with the deviations from the reference cohort at the level of each individual participant.

We define the ‘latent index’ as a feature that indicates the deviation from the normative UMAP of latent variables of each image. To construct this index, we applied normative modeling using a flexible generalization of hierarchical Bayesian regression (HBR) [71–73] to the UMAP of latent variables to remove both linear and non-linear associations with age and sex. Importantly, we used a recent generalization of the HBR method that can handle heteroskedastic and non-Gaussian distributions. Age was defined as a regressor and sex as batch effects. (See de Boer 2022 [73] and S1 File for details of HBR normative model). In this way, for each UMP component of each individual, we obtained the deviation or z-score, which we refer to as the ‘latent index’. Then, we used the latent index as an indicator of individualized brain activation variability by measuring the associations of the latent index and nIDPs using Spearman correlation. To compare this association with linear approaches, we calculated the association between the normative UMAPs of latent variables derived from PCA (number of components = 100) and nIDPs.

## Results

### Autoencoder performance

As described above and in detail in the S1 File, the optimal number of nodes for each layer and the number of layers of semi-supervised AE model were obtained by a pilot study using independent data and resulted 32, 16, and 8 nodes respectively for the 3 layers of the encoder and 8, 16, 32 for the decoder, respectively. Lambda (*λ*) was set empirically at 0.05 in to balance the supervised and unsupervised loss (see S1 File for more details on the architecture of semi-supervised AE and the latent space visualization for different values of lambda). Applying the HCP trained model (without fine-tunning) to UKB data resulted in reconstruction error of 0.31 and 0.17 when *λ* = 0.05 and *λ* = 1, respectively. To elucidate, increasing λ reduced the reconstruction error in the HCP dataset from 0.26 to 0.23, a decrease of 11%. However, this difference was more pronounced in the UKB dataset, where the error reduction was 45%, indicating that lower values of λ significantly impair the autoencoder’s performance not only within the original dataset but also more substantially in a secondary dataset. This shows the latent is not fully generalizable, partially because of age differences between this group in addition to other factors such as demographic differences, differences in signal to noise ratio etc. This motivates retraining the model with UKB data. The out-of-sample performance of the models is shown in Table 1. See S4 Table in S1 File with the extensive comparison with PCA.

**Table 1:** Model performance.

		*HCP*	*UKB*
***Semi-Supervised Autoencoder*** (*λ* = 0.05)			
	Image reconstruction error (MSE)	0.26± 0.00	0.16 ± 0.02
	Age mean absolute error(MAE)	3.13 ± 0.09	4.84 ± 0.25
	Sex prediction accuracy	81% ± 3%	89% ± 3%
***Vanilla Autoencoder*** (*λ* = 1)			
	Image reconstruction error (MSE)	0.23 ± 0.00	0.15 ± 0.01
***PCA***			
	Image reconstruction error (MSE)	0.22 ± 0.00	0.14 ± 0.01

### Visualization of latent space

**Fig 2:**
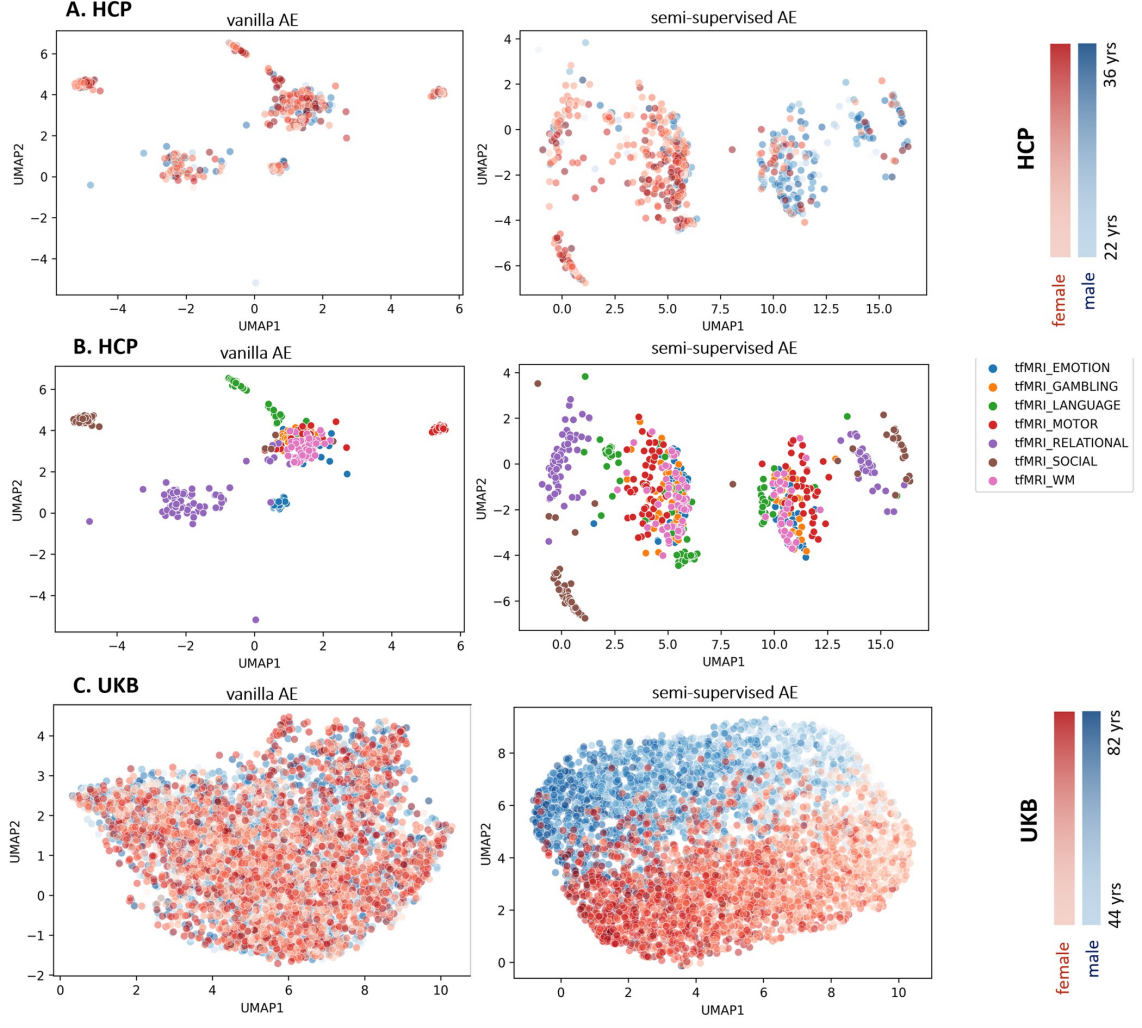
A) UMAP representation of the latent space of *selected contrasts from the Human Connectome Project (HCP) dataset according to Barch 2013*, *colored to show* age and sex separation. B) UMAP representation of the latent space of HCP task contrasts, showing task separation. This is identical to panel A, except that the data points are colored according to task instead of age and sex C) UMAP representation of the latent space of the Faces-Shapes task contrast in the UKB dataset.

The scatterplot of UMAP projections of the autoencoder’s latent variables is shown in Fig 2 for selected contrasts (one per task) [20] in the HCP dataset and the Faces-Shapes contrast from the emotion task in UKB. This figure shows how the data points are distributed in the latent space, indicating the distinctiveness (i.e., functional differentiation) of each task with respect to one another and with regard to age and sex. By contrasting the left and right columns of Fig 2A and 2B, its clear that: (i) in the vanilla AE (*λ* = 1) age and sex are not clearly reflected in the latent space, and rather the latent space principally reflects differences between different tasks; (ii) in the semi-supervised AE (*λ* = 0.05), age and sex are more clearly evident in the latent space, while still providing reasonable separation of the HCP tasks with respect to one another. The relationship with age is especially evident in UKBiobank, where the age range is wider.

### Projection the latent representations to brain images

**Fig 3:**
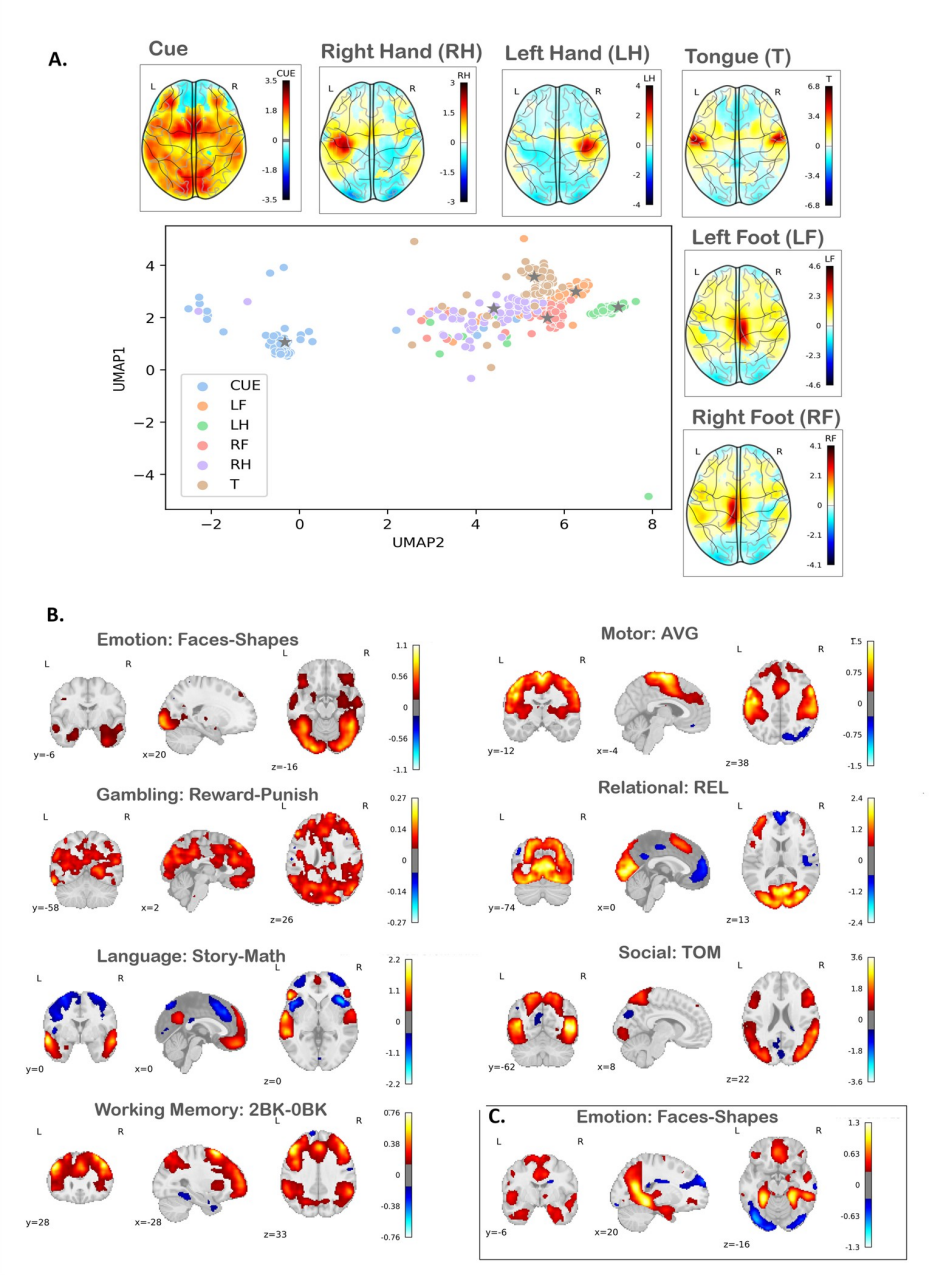
A) **Generated Motor-task contrast**: Changes in the input space activation correspond to moving through the centroid of the latent space for HCP Motor Control subtasks. B) **Generated HCP contrast**: Projections of the centers of the latent representation contrasts (according to Barch 2013) into the input image space. C) **Generated UKB contrast**: Projections of the centers of the latent representation of the Faces-Shapes subtask into the input image space. Abbreviations: LF: left foot; LH: left hand; RF: right foot; RH: right hand; T: tongue.

By navigating through the latent space, we are able to generate various distinct, yet meaningful, image representations and distill topological relationships that may not be evident in the original high dimensional space. This process is akin to moving along a manifold, where each point within this latent space correlates with a unique image and the proximity between points signifies the similarity (or conversely, distinctiveness) of the corresponding images. In this sense, the autoencoder acts as a translator, adeptly converting the complex, high-dimensional input data into a simpler, lower-dimensional, and more comprehensible format, while still preserving topological relationships evident in the high-dimensional data. Fig 3A shows how the activation changes in the input space by moving through the latent space for Motor Control task in HCP. This demonstrates that (i) as expected, the cue condition is the most distinctive from the other contrasts and exhibits high levels of inter-individual variability; (ii) the different motor task contrasts show more of a discrete and mostly more compact representation, with some evidence for higher functional differentiation in the left rather than the right hand. Since most of the HCP participants are right-handed, we interpret this as reflecting a more widespread neural activation pattern for the dominant, relative to the non-dominant hand.

We then map the spatial distribution of the different tasks learned by the autoencoder by projecting the cluster center of several HCP task contrasts (i.e., the main task contrasts reported in Barch et al 2013 [20]) and the Faces-Shapes contrast of UKB to the input space. We compared these synthetic activation patterns (Fig 3B and 3C) with the expected task labels derived from meta-analyses of existing findings using the NeuroSynth [74] meta-analytic database. Table 2 shows the tasks labels having the highest association with previous meta-analytic findings. (See S6 Fig in S1 File for the projection of the center of the UMAP of UKB latent space. While the mapping is not perfect, it is clear that many tasks strongly correspond to the expected cognitive domains (e.g., motor, working memory), although for other tasks the mapping is more ambiguous (e.g., social, gambling).

**Table 2:** Correlation of activations at the center of latent in the original image space with previous findings, as derived from Neurosynth meta-analytic database. Each row represents a specific reported task in existing literature, along with the corresponding correlation values for each subtask in HCP and UKB datasets. Note that the corresponding subtasks per task are: Emotion (Faces-Shapes), Gambling (Reward-Punish), Language (Story-Math), Motor (AVG), Relational (REL), Social (Theory of Mind), and Working Memory (2BK-0BK).

Emotion (HCP)	corr.	Gambling (HCP)	corr.	Language (HCP)	corr.
visual	0.463	memory processing	0.180	social	0.367
face	0.462	decision making	0.176	theory mind	0.334
objects	0.387	visuo-spatial imaginary	0.174	mind	0.298
faces	0.385	mental state	0.135	listening	0.281
**Motor (HCP)**	**corr.**	**Relational (HCP)**	**Corr.**	**Social (HCP)**	**corr.**
motor	0.515	Visual	0.557	visual	0.517
movement	0.488	sensory perception	0.36	motion	0.458
touch	0.484	Task	0.336	object	0.384
voluntary movement	0.443	working memory	0.302	visuospatial cognition	0.374
**Working Memory (HCP)**	**corr.**	**Emotion (UKB)**	**corr.**
working memory	0.384	spatial orientation	0.285
working	0.38	recognition words	0.269
task	0.371	Learning	0.176
inhibitory control	0.37	visual information	0.159

### Association between latent variables and non-imaging covariates

**Fig 4:**
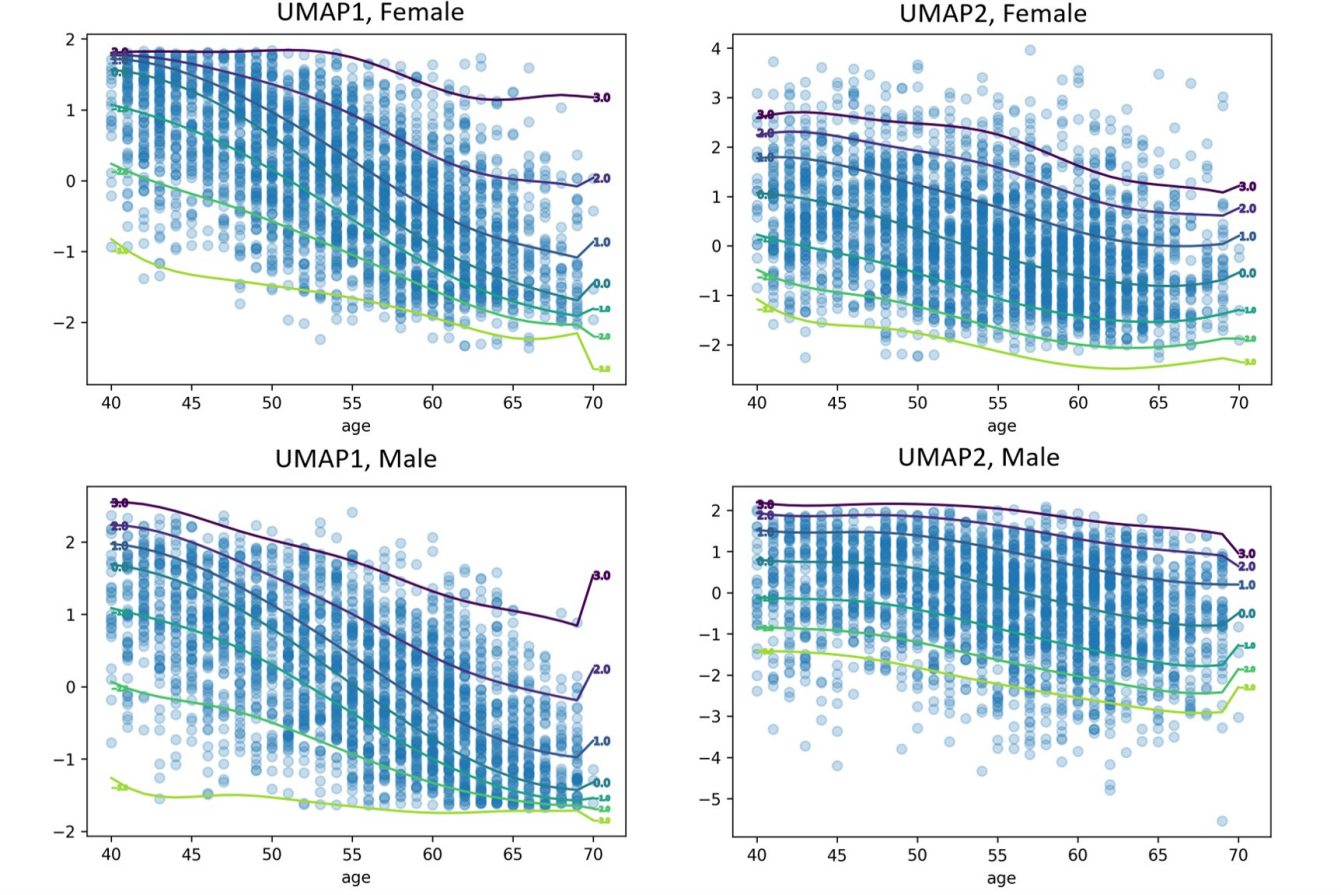
Normative models of the latent space UMAP components for males and females. The figure presents four separate normative models for two UMAP components of the latent space, each depicting the relationship between age and the corresponding UMAP component. The individualized deviations from the normative range represent the latent representation index. Each percentile line within each figure displays the level of deviation from the normative range for each time point, illustrating the degree to which individuals differ from the expected normative pattern across various percentiles.

Next, we aim to show the utility of the latent space in providing biomarkers on the basis of normative models. Fig 4 displays normative models applied to the UMAP representation of latent variable (see S7 Fig in S1 File for measures of fit for the HBR model). The distribution of the UMAP representations have a complex and non-Gaussian distribution (See qq-plots in S7 Fig in S1 File), which can be effectively modeled by leveraging the flexibility of HBR model. For example, in the left panels the distribution is negatively skewed for younger ages but becomes positively skewed for ages increase.

**Fig 5:**
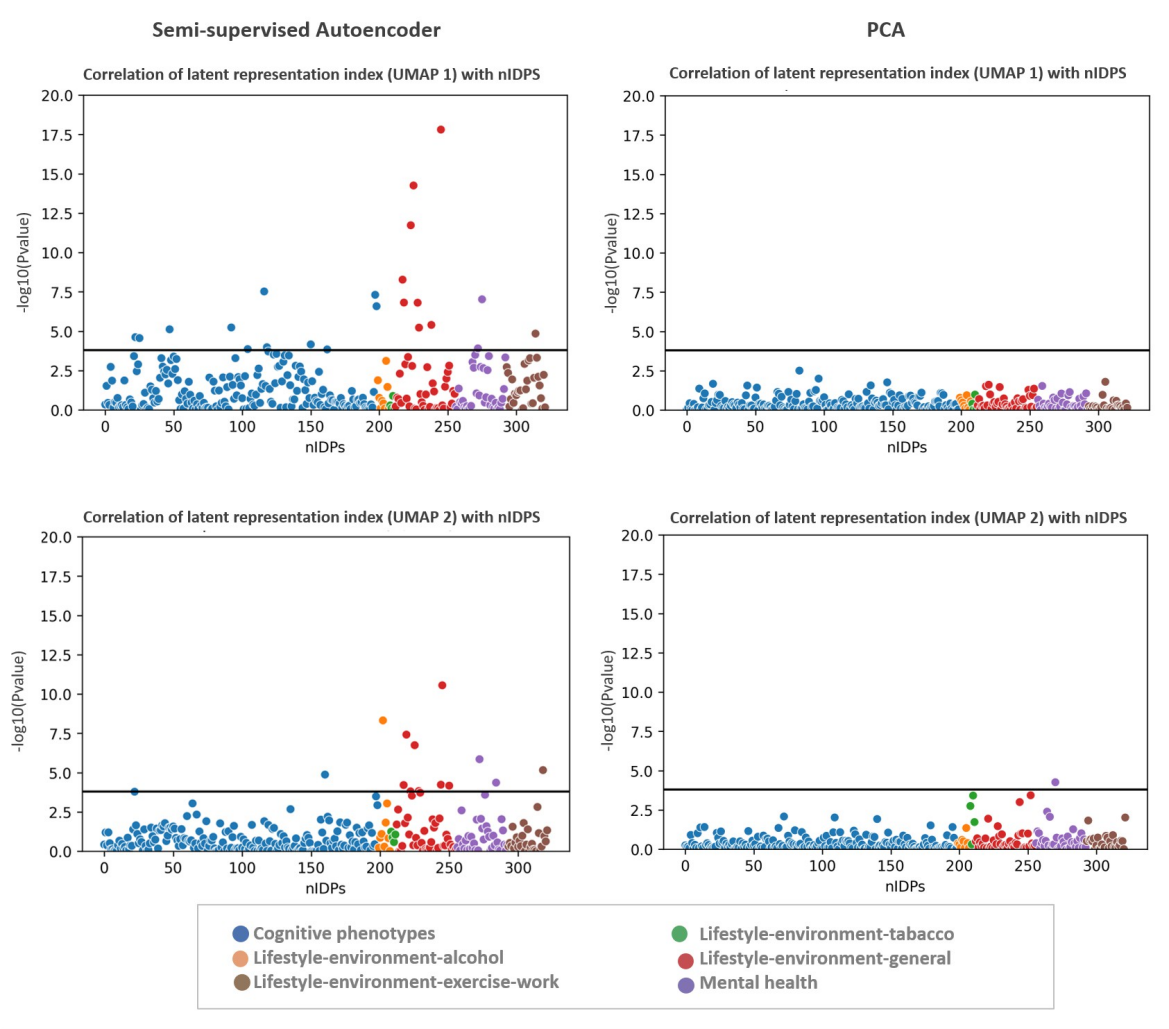
Manhattan plot of p-value of univariate correlation of non-imaging measures with the individualized deviations from normative UMAPs of latent space (latent representation index) and PCA. The black line is Bonferroni-corrected p-value threshold.

Fig 5 shows the Manhattan plot of the p-value of univariate correlation between non-imaging measures and the deviations from the normative model fit on the latent index of semi-supervised AE and PCA. This indicates strong associations with many nIDPs even after properly accounting for age and sex using the normative model. Furthermore, these associations are considerably stronger using semi-supervised autoencoder compared to the unsupervised representation provided by PCA. See S8 Fig in S1 File for the effect size of the associations between nIDPs and the latent index. We show the correlation of the raw UMAP scores with nIDPs in S9 Fig in S1 File (i.e. without first fitting a normative model to the latent variables). These associations are also strong but we consider this result with caution because it is potentially partially due to the high level of correlation between age, sex, and other cognitive and behavioral measures. Rather, we consider it preferable to interpret the deviations from a model that first removes the confounding effects of age and sex from the latent variables through the application of normative modeling.

Finally, we also performed comparisons of our approach with several alternative baseline methods, namely (i) a vanilla PCA on the image data, (ii) a two-stage PCA procedure that includes age and sex, thereby aiming to provide a linear benchmark for the semi-supervised autoencoder (iii) independent components analysis (ICA) and (iv) anatomically defined regions of interest. In summary, these approaches all resulted in considerably poorer performance and weak associations with non-imaging measures (see S4 Table and S10-S13 Figs in S1 File).

## Discussion

In this study, our primary objective was to develop a 3-D convolutional autoencoder architecture capable of extracting more informative, lower-dimensional latent representations of brain activity from task-based fMRI data. These representations better capture the underlying neural processes associated with specific cognitive tasks and demonstrate the potential to predict behavioral and cognitive measures. While the concept of lower-dimensional representations and their relation to behavior is not novel, our work contributes by tailoring the autoencoder-based model to fMRI data, providing interpretable latent representations that can be compared to linear methods like PCA. Importantly, through our joint optimization framework, we are able to control the representations that the autoencoder learns, in this case, learning a manifold related to age and sex, where the deviations from this manifold are informative about individual differences and provide candidate biomarkers. By "interpretable" and "meaningful latent representations," we refer to the ability of these representations to be effectively mapped to non-imaging variables and to be understood both within the latent space and in the original voxel space. This dual interpretability ensures that the latent space not only captures essential features relevant to the imaging data but also maintains a clear relationship with biological and clinical variables. As a result, decoding a latent representation yields outputs that are coherent with the original data, ensuring that the generated outputs are biologically plausible and clinically relevant. We argue that this capability is essential for validating the model’s predictions and ensuring that the latent representations are effective for further analyses, such as predicting cognitive or behavioral outcomes.

Our model allows for accurate reconstruction of the data while representing demographic variation and presents methods to visualize, interpret, and control the learned latent space representation. We show a simple example of how this approach can be used to understand topological relationships of different task representations in the brain using the HCP motor task. By defining a latent index, we established the utility of this approach for developing biomarkers that predict behavioral measures. We demonstrated that our model learns salient features that capture age, other sources of population stratification, and are strongly associated with clinical and behavioral features.

### Learning a generic latent representation

While our approach can be applied to various types of imaging data, we chose to focus on task-based fMRI contrasts in this study. Task-based fMRI is easily relatable to specific cognitive functions and has a long history in the neuroimaging field as suitable method for studying associations between brain and behavior. However, our approach could also be adopted for other types of neuroimaging-derived readouts, although this might require a different architecture than the one we used here. More specifically, the HCP task-fMRI data enabled us to estimate a generic latent space representation across diverse cognitive tasks [19, 20], whilst also providing good whole brain coverage across all the tasks [20]. During the training, this mapping allows the autoencoder to learn various activation patterns across the brain, rather than focusing on specific task-related effects that might be localized to particular brain regions.

### Mapping the latent space

Considering the limited age range and the relatively small number of test participants in each HCP model (N≈95), the effect of age and sex in the latent space is not clear, although the relationships between tasks (Fig 2B) and different conditions within the same task (Fig 2A) are clearly distinguishable. In contrast, the UMAP of UKB generates a clear age continuum and good separation in terms of sex. This indicates that moving from one point in the manifold to another can be meaningfully traced back through the input space and that changes within the latent space reflect salient changes within the input data, although the precise relationships may be different across datasets, depending on the degree to which different modeled variables are reflected in the original data. This is in line with the standard interpretation of autoencoders as learning a manifold [49].

Unsupervised training of the model yields interpretable representations where different task contrasts are well-clustered and separated in the latent space. In contrast, with the semi-supervised learning, our representation is tailored to focus on mapping the latent space back to the original space, with the added control of age and sex. This approach ensures that the latent manifold clearly reflects individual differences related to the demographic variables in the underlying imaging data, whilst still providing good separation in terms of task contrasts.

Projection of latent representation to original space: For the majority of the contrasts and particularly language (story-math), working memory (2BK-0BK), and motor control (AVG), the projection of the center of the latent space to the original scan image space faithfully represents the group maps in [20]. In the context of interpretability of findings, the meaningful projection of the latent space can be viewed as an example of explainable AI in complex models.

### Association of the latent representation index with nIDPs measures

A key aspect of summarizing the complex spatial maps of tfMRI is to preserve individual variability. To complement this, these summaries or representations should contain biological information that can be linked to cognitive, behavioral and clinical characteristics. Since the latent space also represents age and sex, and because age is strongly associated with a variety of cognitive and behavioral scores, the correlation of latent variables and nIDPs may be disrupted by the confounding effect of age (see S8 Fig in S1 File for the correlation of UMAPs and nIPDs). To disentangle clinically relevant variation from variation due to age and sex from the UMAP representation, we applied normative modeling based on hierarchical Bayesian regression. Here, the individualized deviations or latent representation index indicates the distance from the normative latent variables transformed by UMAP. We showed that this index is strongly associated with several nIDP scores after accounting for confounding variables (age and sex). Hence, the notion of normative latent variables may provide the basis for the development of a biomarker that predicts cognitive and behavioral characteristics. Moreover, we show that this association is stronger than classical linear models such as PCA.

### Network architecture

The architectural hyper-parameters of the autoencoder were chosen during the pilot study, solely based on model’s performance in terms of the reconstruction error; no other readouts i.e., non-imaging measures, were used for evaluations. Additionally, the data used for the pilot study were not reused. Some decisions about the network structure were made before estimating the model. For example, to preserve the morphology of the images and thus improve the interpretability, we decided to use a 3-D convolutional network [30–32, 45]. In order to control over of latent space, we used a dense layer in the bottleneck of the autoencoder [49].

We designed our autoencoder with the specific nature of our high-dimensional neuroimaging data in mind, imposing several constraints on the model beforehand. For example, the networks evaluated were not particularly deep. To reduce the memory usage and computational complexity, we took advantage of the weight sharing of convolutional layers. We aimed to identify low-level features that may be translation invariant, with a significant benefit being the ability to scale the networks to whole-brain data [75]. The kernel size was set to be 3×3×3 to retain the details of the downsampled image scans. Average pooling layers were positioned after each convolutional layer to smooth sharp features, reduce the number of parameters, and minimize the chance of overfitting. We relied on the pilot study to select the remaining model parameters, such as the number of filters.

We assigned both unsupervised (image reconstruction error) and supervised (age and sex prediction) loss functions to our semi-supervised AE, aiming to find meaningful latent representations of data that can be mapped to the non-imaging variables and interpreted both in the latent space and in the original voxel space. Our model showed high performance in predicting age and sex. The contribution of supervised and unsupervised loss can be also redefined in order to emphasize the optimization process. This results in a semi-supervised setting that allows the latent space to partially encode specific features of the data [8]. Another interesting future direction is to train an autoencoder to predict different data (e.g., a follow-up timepoint in longitudinal studies). This would sensitize the latent space to changes relevant to aging or pathology, suggesting that the latent representation may also be useful for generating features for downstream analyses aiming at predicting these changes.

### Limitations and future work

The increased number of neuroimaging scans provides a unique opportunity to transcend linear mappings, but it is also necessary to acknowledge some limitations. Traditional image processing techniques often used in deep learning are not entirely applicable here. For example, while data augmentation methods such as image mirroring, flipping, skewing, or segmenting are straightforward approaches to increase the number of samples and have been applied in neuroimaging applications [11], we did not consider them appropriate here. Such augmentation strategies do not faithfully preserve invariances known to occur in the brain, such as the lateralization of brain functions (e.g. the association of left lateralization in language processing [76]).

The generalizability of the latent representation is another important concern that has not been fully addressed here due to age range differences between two datasets. UKB contains the Hariri faces-shapes emotion task [77], which is very similar to the emotion task of HCP (effectively a shorter version). Although the common contrasts provide a great opportunity for further validation of the model, the age gap limits the capacity to test the generalization of the latent space across cohorts.

Computational complexity is another limitation. Training an autoencoder on large neuroimaging data is computationally more demanding compared to linear models. In this work, we set the trade-off parameter (lambda) governing the contribution of supervised and unsupervised loss components in a relatively informal manner. A quantitative evaluation would have required us to define the relative value of each component (e.g. how much to favor prediction of the supervised targets over reconstruction or vice versa). It is possible that more careful optimization of this parameter may yield improved performance.

In our study, we focused on age and sex as supervised factors in our model, as they are well-known demographic variables with significant effects on brain structure and function. By incorporating these factors, we aimed to disentangle the age- and sex-related variations from other sources of variability in the data. However, we acknowledge that there may be other factors contributing to the differences between the HCP and UK Biobank samples, such as socioeconomic status, education, and genetic predispositions. These factors could also influence the latent representations and their generalizability. In future work, it would be valuable to extend our model to incorporate additional factors and investigate how they contribute to the observed inter-individual differences in brain activity. This extension will help provide a more comprehensive understanding of the factors influencing the generalizability of the latent representations and their relationships with brain-behavior associations.

Furthermore, we recognize the importance of comparing our approach to alternative methods and demonstrating its potential applicability in clinical practice. Our study compares our model to PCA, ICA, and ROIs , showing that the autoencoder-based approach better predicts behavioral and cognitive measures. We also believe our model holds promise for clinical practice, as it may help identify atypical patterns of brain activity not easily detected by traditional behavioral measures. This could be particularly relevant for large-scale datasets like the UK Biobank, where neuroimaging data can be leveraged to inform personalized interventions and improve clinical outcomes, ultimately advancing our understanding of the links between the brain and behavior.

In this work we principally used the very low-level representation derived from the UMAP, however for some analyses it may be preferable to use the ultimately richer representation derived from the autoencoder itself. Such an approach may be particularly valuable to relate the learned representation to external variables using multivariate methods such as classifiers or canonical correlation analysis.

## Conclusion

Here, we applied 3-dimensional autoencoder to two large-scale datasets to find an interpretable latent representation of high dimensional task fMRI image data by controlling for demographic information. We applied normative modeling to the latent variables to define an index to find a mapping to non-imaging measures.

Our model showed high performance in terms of age and sex prediction and was capable of capturing complex biological, cognitive, and clinical characteristics while preserving the individualized variabilities using a latent representation index. We consider this representation to provide an excellent basis for understanding inter-individual differences in neural representations in clinical and fundamental neuroscience research.

## Supporting information

S1 File (PDF)
