# Supplementary material for: Nonlinear latent representations of high-dimensional task-fMRI data: Unveiling cognitive and behavioral insights in heterogeneous spatial maps
Source: PLoS One. 2024 Aug 8;19(8):e0308329. doi: 10.1371/journal.pone.0308329 (PMC11309387; doi:10.1371/journal.pone.0308329)
Supplement: S1 File — (PDF) [file pone.0308329.s001.pdf]

Supplementary Materials

Nonlinear Latent Representations of High-Dimensional Task-fMRI Data: Unveiling Cognitive and Behavioral Insights in Heterogeneous Spatial Maps

Mariam Zabihi[1,2,3], Seyed Mostafa Kia[1,2,4], Thomas Wolfers[1,2,5,6], Stijn de Boer[1], Charlotte Fraza[1,2], Richard Dinga[1,2], Alberto Llera Arenas[1], Danilo Bzdok[7,8], Christian F. Beckmann[1,2,9], Andre Marquand[1,2,10]

[1]Donders Institute for Brain, Cognition and Behavior, Radboud University Nijmegen, Nijmegen, the Netherlands
[2]Department for Cognitive Neuroscience, Radboud University Medical Center Nijmegen, Nijmegen, the Netherlands
[3]MRC Unit for Lifelong Health & Ageing, University College London (UCL), London, United Kingdom
[4]Department of Psychiatry, University Medical Center Utrecht, Utrecht, the Netherlands
[5]NORMENT, KG Jebsen Centre for Psychosis Research, Division of Mental Health and Addiction, Oslo University Hospital & Institute of Clinical Medicine, University of Oslo, Oslo, Norway
[6]Department of Psychiatry and Psychotherapy, Tübingen Center for Mental Health, University of Tübingen, Tübingen, Germany
[7]Multimodal Imaging and Connectome Analysis Lab, McConnell Brain Imaging Centre, Montreal Neurological Institute and Hospital, McGill University, Montreal, Quebec, Canada
[8]Mila - Quebec Artificial Intelligence Institute, Montreal, Quebec, Canada
[9]Centre for Functional MRI of the Brain, University of Oxford, Oxford, United Kingdom
[10]Department of Neuroimaging, Institute of Psychiatry, Psychology, & Neuroscience, King's College London, London, United Kingdom

1-Methods

1-1 Data:

The age distributions of HCP[1] and UK-biobank[2] tfMRI data across sex are shown in S1 Fig.

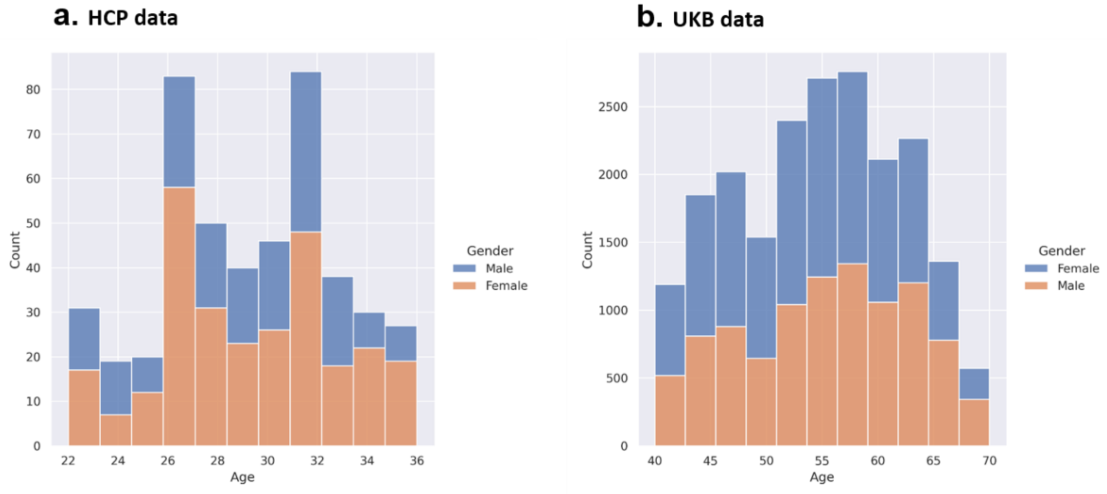

S1 Fig: Age distribution in a) HCP and b) UK biobank

The list of UK-bio bank behavioral measures categories[2] is shown S1 Table.

S1 Table: UK biobak non-imaging variable list

Non-imaging variables categories
Basic demographic
Cognitive phenotypes
Lifestyle environment alcohol
Lifestyle environment tobacco
Lifestyle environment general
Lifestyle environment exercise work
Mental health
Age, sex, site

1-2 Model training:

All the models were trained using NVIDIA P100 GPU, TensorFlow r2.8. The training time for HCP data with N=32K scans was approximately 11.5 minutes per epoch. The fine-tuning time for UKbiobank with N=15K was 2 minutes per epoch. The batch size was 10 for all the models.

1-3 Semi-Supervised AE

The architecture of 3D semi-supervised AE is shown in S2 Fig. We used 2580 scans from HCP (N=30 participants) for selecting the architecture and hyperparameters of the network. These scans never used again in the training and test phases.

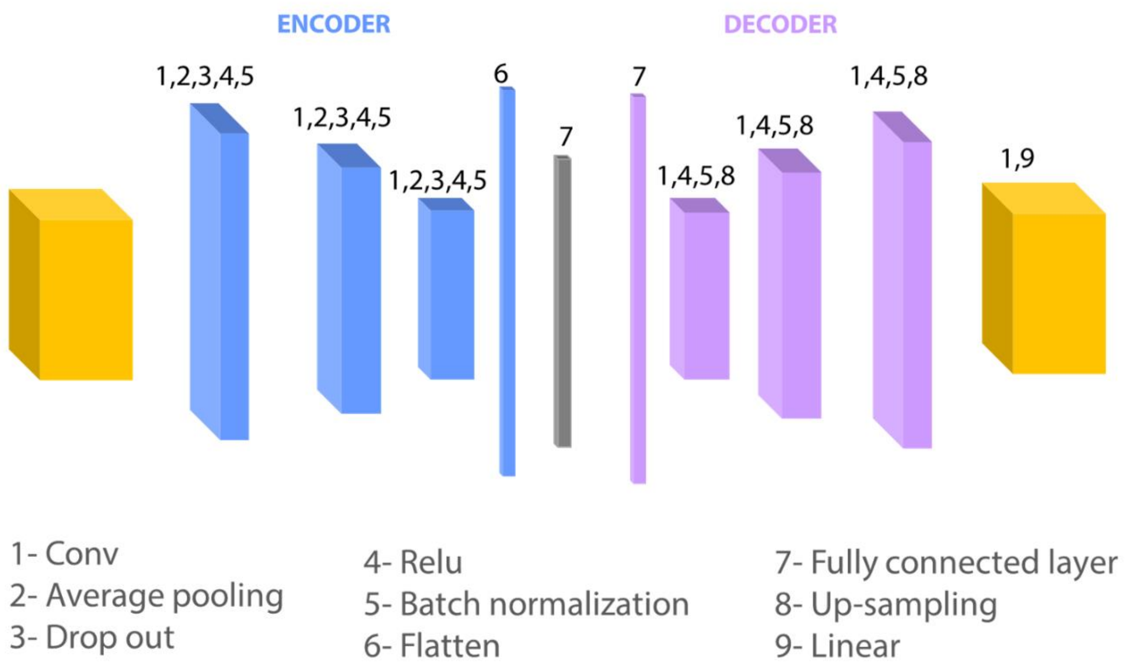

S2 Fig: Autoencoder architecture

1-4 UMAP hyper-parameters

We used the default parameters of UMAP for training. We tuned the number of neighbors (n_neighbor=15) and minimum distance of the points (min_dist=.1) in a cluster to maintain the balance between the global and local structure of data. Moreover, we selected Euclidean distance for computing the ambient space of data. These parameters provided robust results and better visual separation.

1-5 Normative modeling

Briefly, normative modeling presents a probabilistic interpretation of the deviations of the latent variables (UMAP projections) across all subjects. To measure the deviations, we used the predicted UMAP projections of the normative model for each individual participant, and next converted each to a subject-specific *Z* score as described previously[3].

We applied Hierarchical Bayesian Regression (HBR) to perform normative modeling. Here, we modeled the posterior probability of the brain data, Y, given X, i.e. P(Y|X) using MCMC sampling. For the data likelihood, we used a sinh-arcsinh (SHASH) distribution (Jones and Pewsey, 2009[4]), which is derived from passing a Gaussian distributed random variable, x, through the transformation: $\sinh((\sinh^{-1}(x) + \gamma)/\kappa))$, where the parameters γ and κ govern shape of the underlying distribution. We and others have shown that this distribution is well-suited to modelling non-Gaussian data as it can model both platykurtic and leptokurtic distributions and positive and negative skew in the same distributionally family[4–6]. We used a specific reparameterization of this distribution, referred to as SHASHb, which breaks the dependency between shape parameters, such that γ and κ relate to the skew and kurtosis respectively (see de Boer et al 2022 for details). To model non-linearity in the mean, variance and skew, we used a cubic b-spline basis expansions with. 5 equally spaced knot points. To further increase the flexibility of the model, we added a random effect of sex to the mean, variance, skew parameters.

So briefly, our model is given by:

1.Likelihood probability with SHASHb distribution:

$$P(Y|\mu, \sigma^2, \gamma, \kappa) = SHASHb(Y; \mu, \sigma^2, \gamma, \kappa)$$

where:

Y = observed data

μ = mean

σ^2 = variance

γ = skew

κ = kurtosis

2.Regression model with B-spline basis expansion:

$$\mu_i = f(X_i) = \sum B_j(X_i) * \beta_j$$

where:

μ_i = mean for observation *i*

$f(X_i)$ = B-spline basis expansion of the regression

$B_j(X_i)$ = B-spline basis function *j* evaluated at X_i

β_j = regression coefficient for B-spline basis function *j*

3.Random-effects model for mean, variance, and skew parameters:

$$\mu_i = f(X_i) + u_i$$

$$\sigma^2_i = g(X_i) + v_i$$

$$\gamma_i = h(X_i) + w_i$$

where:

u_i ,v_i ,w_i = random effects for mean, variance, and skew parameters, respectively

$f(X_i)$, $g(X_i)$, $h(X_i)$ are B-spline basis expansions over age for mean, variance, and skew parameters, respectively

Inference was performed using Markov chain Monte Carlo inference, using the no U-turn (NUTS) sampler implemented in the pymc3 python package. Note that these equations provide only a brief overview of the HBR model described above. The full details of this approach can be found in [de Boer 2022][5] and the HBR implementation is provided in the pcntoolkit package (https://github.com/amarquand/PCNtoolkit).

1-6 Comparison with mixed-PCA

In addition to standard PCA, we perform an additional analysis that aimed to also include age and sex in the model to mimic a linear variant of the semi-supervised approach. This helps to understand the contributions of the nonlinear nature of the autoencoder and the inclusion of age and sex. To do so, we conducted a simple 2 stage PCA that accommodate for age and sex in the second stage of PCA. First, we applied PCA to the data, resulting in N components. Then, we added age and sex as separate components to the N PCA components and applied another PCA to reduce the dimensionality to M components ($M<N$). This process allowed us to mix the PCA components linearly with age and sex information.

1-7 Comparison with ICA

We applied sklearn's FastICA to vectorized data with 100 components. Following this, we applied UMAP to the ICA components, and then used the transformed UMAP components to calculate the univariate correlation with non-imaging-derived phenotypes (nIDPs).

1-8 Comparison with (Region of interests) ROIs

The underlying assumption here is that ROIs are hand-crafted summaries of the data. Similar to the approach with ICA components, we applied UMAP and measured the univariate correlations.

2- Results

2-1 Model selection: Hyper-parameters tuning

S2 Table shows the performance of the models with different configurations and image normalization strategies in terms of normalized root mean square of reconstruction error (NRMSE), which is the root mean squared error scaled by the range of the input image.

S2 Table: Comparison model performance for different parameters

nmse	optimizer	dropout	encoder_filters	decoder_filters	lr	scaler_type	dense_layer
0.028	RMSprop	0.2	[32, 16, 8]	[8, 16, 32]	0.001	Sandard_Feature-wise	50
0.033	RMSprop	0	[32, 16, 8]	[8, 16, 32]	0.001	MinMax_Feature-wise	10
**0.027**	**RMSprop**	**0.2**	**[32, 16, 8]**	**[8, 16, 32]**	**0.001**	**Sandard_Feature-wise**	**100**
0.031	RMSprop	0	[32, 16, 8]	[8, 16, 32]	0.001	MinMax_Feature-wise	50
0.033	RMSprop	0	[32, 16, 8]	[8, 16, 32]	0.001	Sandard_Feature-wise	10
0.030	RMSprop	0	[32, 16, 8]	[8, 16, 32]	0.001	Sandard_sample-wise	10
0.032	RMSprop	0	[32, 16, 8]	[8, 16, 32]	0.001	Sandard_Feature-wise	50
0.028	RMSprop	0	[32, 16, 8]	[8, 16, 32]	0.001	Sandard_Feature-wise	100
0.030	RMSprop	0.2	[32, 16, 8]	[8, 16, 32]	0.001	Sandard_Feature-wise	10
0.031	RMSprop	0	[32, 16, 8]	[8, 16, 32]	0.001	MinMax_Feature-wise	100
0.035	RMSprop	0	[32, 16, 8]	[8, 16, 32]	0.001	Sandard_sample-wise	50
0.032	RMSprop	0.2	[32, 16, 8]	[8, 16, 32]	0.001	MinMax_Feature-wise	10
0.036	RMSprop	0	[32, 16, 8]	[8, 16, 32]	0.001	Sandard_sample-wise	100
0.031	RMSprop	0.2	[32, 16, 8]	[8, 16, 32]	0.001	MinMax_Feature-wise	50
0.034	RMSprop	0.2	[32, 16, 8]	[8, 16, 32]	0.001	Sandard_sample-wise	10
0.030	RMSprop	0.2	[32, 16, 8]	[8, 16, 32]	0.001	MinMax_Feature-wise	100
0.039	RMSprop	0.2	[32, 16, 8]	[8, 16, 32]	0.001	Sandard_sample-wise	50
0.042	RMSprop	0.2	[32, 16, 8]	[8, 16, 32]	0.001	Sandard_sample-wise	100
0.032	RMSprop	0	[8, 16, 32]	[32, 16, 8]	0.001	Sandard_sample-wise	10
0.037	RMSprop	0	[8, 16, 32]	[32, 16, 8]	0.001	Sandard_sample-wise	50
0.038	RMSprop	0	[8, 16, 32]	[32, 16, 8]	0.001	Sandard_sample-wise	100
0.033	RMSprop	0	[8, 16, 32]	[32, 16, 8]	0.001	MinMax_Feature-wise	10
0.037	RMSprop	0.2	[8, 16, 32]	[32, 16, 8]	0.001	Sandard_sample-wise	10
0.032	RMSprop	0	[8, 16, 32]	[32, 16, 8]	0.001	MinMax_Feature-wise	50
0.036	RMSprop	0.2	[8, 16, 32]	[32, 16, 8]	0.001	Sandard_sample-wise	50
0.031	RMSprop	0	[8, 16, 32]	[32, 16, 8]	0.001	MinMax_Feature-wise	100
0.036	RMSprop	0.2	[8, 16, 32]	[32, 16, 8]	0.001	Sandard_sample-wise	100
0.032	RMSprop	0.2	[8, 16, 32]	[32, 16, 8]	0.001	MinMax_Feature-wise	10
0.140	RMSprop	0	[32, 16, 8]	[8, 16, 32]	0.001	MinMax_sample-wise	10
0.031	RMSprop	0.2	[8, 16, 32]	[32, 16, 8]	0.001	MinMax_Feature-wise	50
0.162	RMSprop	0	[32, 16, 8]	[8, 16, 32]	0.001	MinMax_sample-wise	50

0.065	RMSprop	0	[32, 16, 8]	[8, 16, 32]	0.001	MinMax_sample-wise	100
0.104	RMSprop	0.2	[32, 16, 8]	[8, 16, 32]	0.001	MinMax_sample-wise	10
0.031	RMSprop	0.2	[8, 16, 32]	[32, 16, 8]	0.001	MinMax_Feature-wise	100
0.188	RMSprop	0.2	[32, 16, 8]	[8, 16, 32]	0.001	MinMax_sample-wise	50
0.165	RMSprop	0.2	[32, 16, 8]	[8, 16, 32]	0.001	MinMax_sample-wise	100
0.084	RMSprop	0	[8, 16, 32]	[32, 16, 8]	0.001	MinMax_sample-wise	10
0.063	RMSprop	0	[8, 16, 32]	[32, 16, 8]	0.001	MinMax_sample-wise	50
0.029	RMSprop	0	[8, 16, 32]	[32, 16, 8]	0.001	Sandard_Feature-wise	10
0.083	RMSprop	0	[8, 16, 32]	[32, 16, 8]	0.001	MinMax_sample-wise	100
0.116	RMSprop	0.2	[8, 16, 32]	[32, 16, 8]	0.001	MinMax_sample-wise	10
0.070	RMSprop	0.2	[8, 16, 32]	[32, 16, 8]	0.001	MinMax_sample-wise	50
0.080	RMSprop	0.2	[8, 16, 32]	[32, 16, 8]	0.001	MinMax_sample-wise	100
0.028	RMSprop	0	[8, 16, 32]	[32, 16, 8]	0.001	Sandard_Feature-wise	50
0.028	RMSprop	0	[8, 16, 32]	[32, 16, 8]	0.001	Sandard_Feature-wise	100
0.030	RMSprop	0.2	[8, 16, 32]	[32, 16, 8]	0.001	Sandard_Feature-wise	10
0.028	RMSprop	0.2	[8, 16, 32]	[32, 16, 8]	0.001	Sandard_Feature-wise	50
0.027	RMSprop	0.2	[8, 16, 32]	[32, 16, 8]	0.001	Sandard_Feature-wise	100
0.029	RMSprop	0	[32, 16, 8]	[8, 16, 32]	0.01	Sandard_Feature-wise	100
0.032	RMSprop	0	[32, 16, 8]	[8, 16, 32]	0.005	Sandard_Feature-wise	100
0.034	RMSprop	0	[32, 16, 8]	[8, 16, 32]	0.0001	Sandard_Feature-wise	100
0.029	Adam	0	[32, 16, 8]	[8, 16, 32]	0.01	Sandard_Feature-wise	100
0.028	Adam	0	[32, 16, 8]	[8, 16, 32]	0.001	Sandard_Feature-wise	100
0.052	Adam	0	[32, 16, 8]	[8, 16, 32]	0.0001	Sandard_Feature-wise	100

2-2 The impact of supervised loss coefficient λ on latent

To selection, we trained the model with different values of $\boldsymbol{\lambda}$. S3 Table indicates the impact of $\boldsymbol{\lambda}$ on unsupervised and supervised loss in the small held-out test data set of HCP. As expected, $\boldsymbol{\lambda} = 1$ fails to predict age and sex. By increasing the supervised loss impact and decreasing $\boldsymbol{\lambda}$, however, the reconstruction loss increases.

$\boldsymbol{\lambda} = 0.05$ shows the better performance in terms of sex and age prediction and reconstruction error, so we use this value for all the main analyses reported in the main text.

S3 Table: model performance in terms of loss values for different values of λ

λ	Reconstruction error	Balanced accuracy	Mean Absolute Error [years]	Total loss
**1**	0,120	0,551	29,075	29,884
**0,995**	0,125	0,966	1,275	1,564
**0,95**	0,128	0,996	0,963	1,101
**0,5**	0,188	0,996	0,995	1,190
**0,05**	0,193	1,000	0,820	**1,016**
**0,005**	0,191	1,000	1,013	1,207
**0,0005**	0,192	0,994	0,833	1,043

S3 Fig Shows how the change of λ reflects on latent space in UKB.

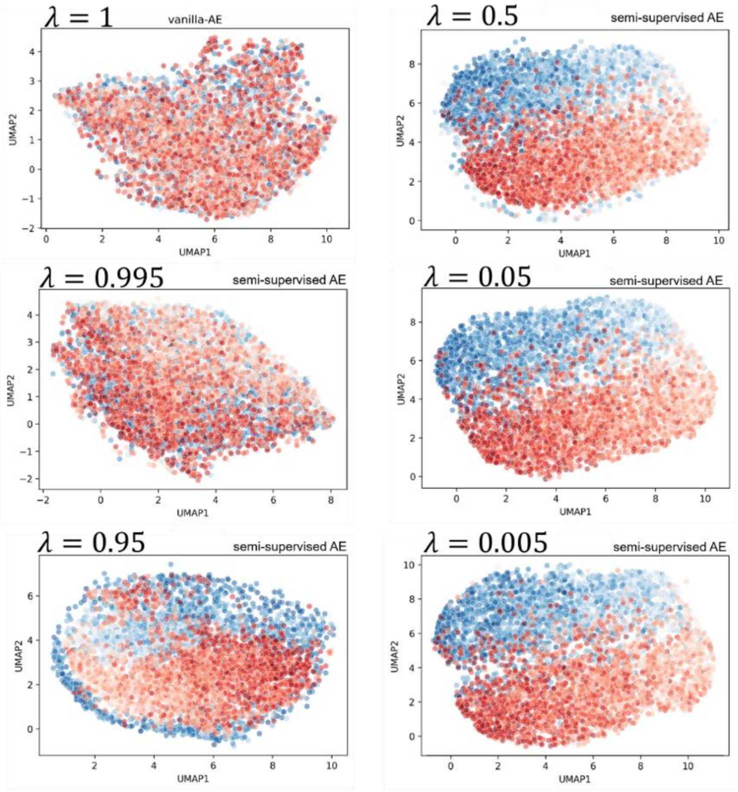

S3 Fig: change of latent space representation by changing λ

2-3 Autoencoder learning curve

The learning curve of semi-supervised AE is shown in S4 Fig.

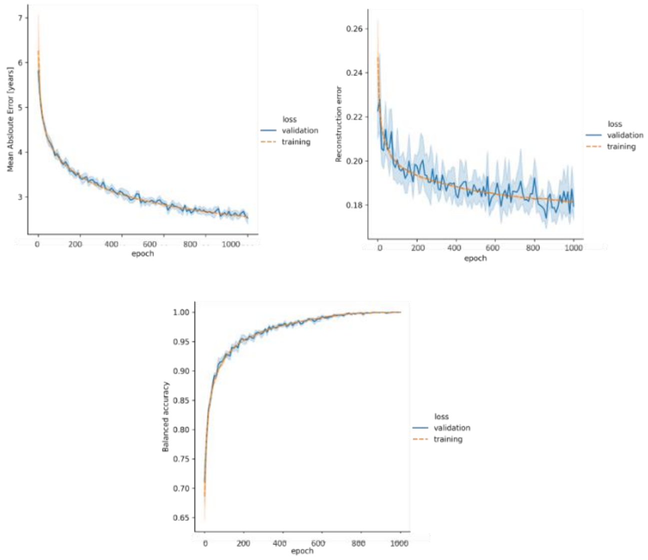

S4 Fig: The mean learning curve of semi-supervised AE using UKB

2-4 Autoencoder learns different level features in each layer

S5 Fig shows the weight of kernel in several layers. This shows that the autoencoder learns complex features of the data (and not just the identity function).

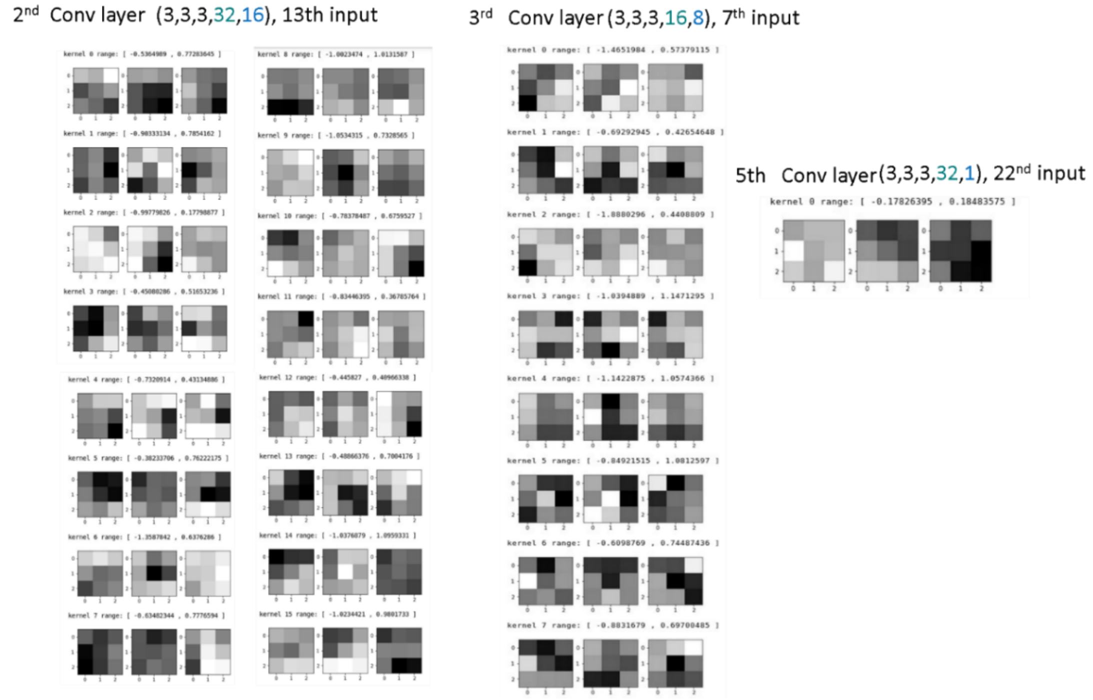

S5 Fig: Kernels's weights for selected layers

2-5 Comparison with PCA with age and sex:

We extend the PCA comparison to emphasis that behavioral and cognitive properties are not reflected in linear representation and evaluated different number of components. S4 Table shows the mean absolute error of reconstruction among the PCAs models. N and M are the number of first and second PCA components, respectively. (M<N)

S4 Table: :PCA model performance for UKB

N (M) PCA components	Mean Square Error (MSE)
4(2)	0.67
7(5)	0.56
12(10)	0.52
102(100)	0.41
502(500)	0.32
1002(1000)	0.26

2-6 Projection the latent representations to brain images for UKB

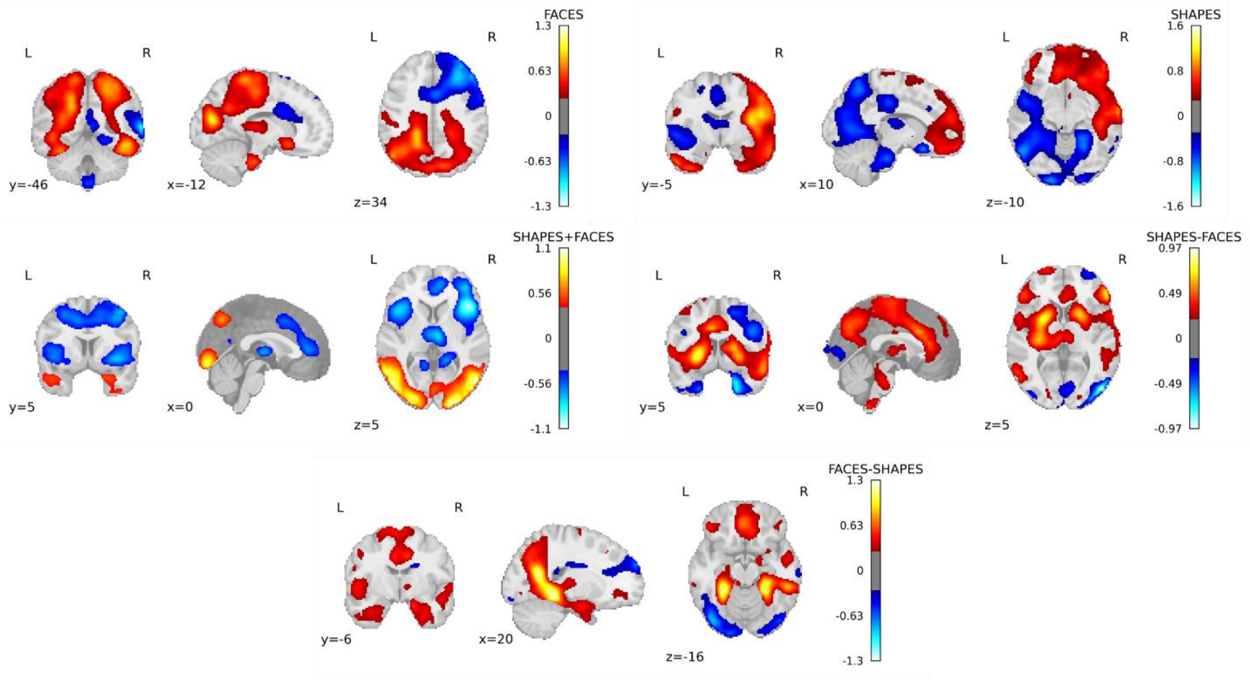

S6 Fig: The projection of centroid of UMAP in the latent space to the input image space.

2-7 HBR normative modelling performance

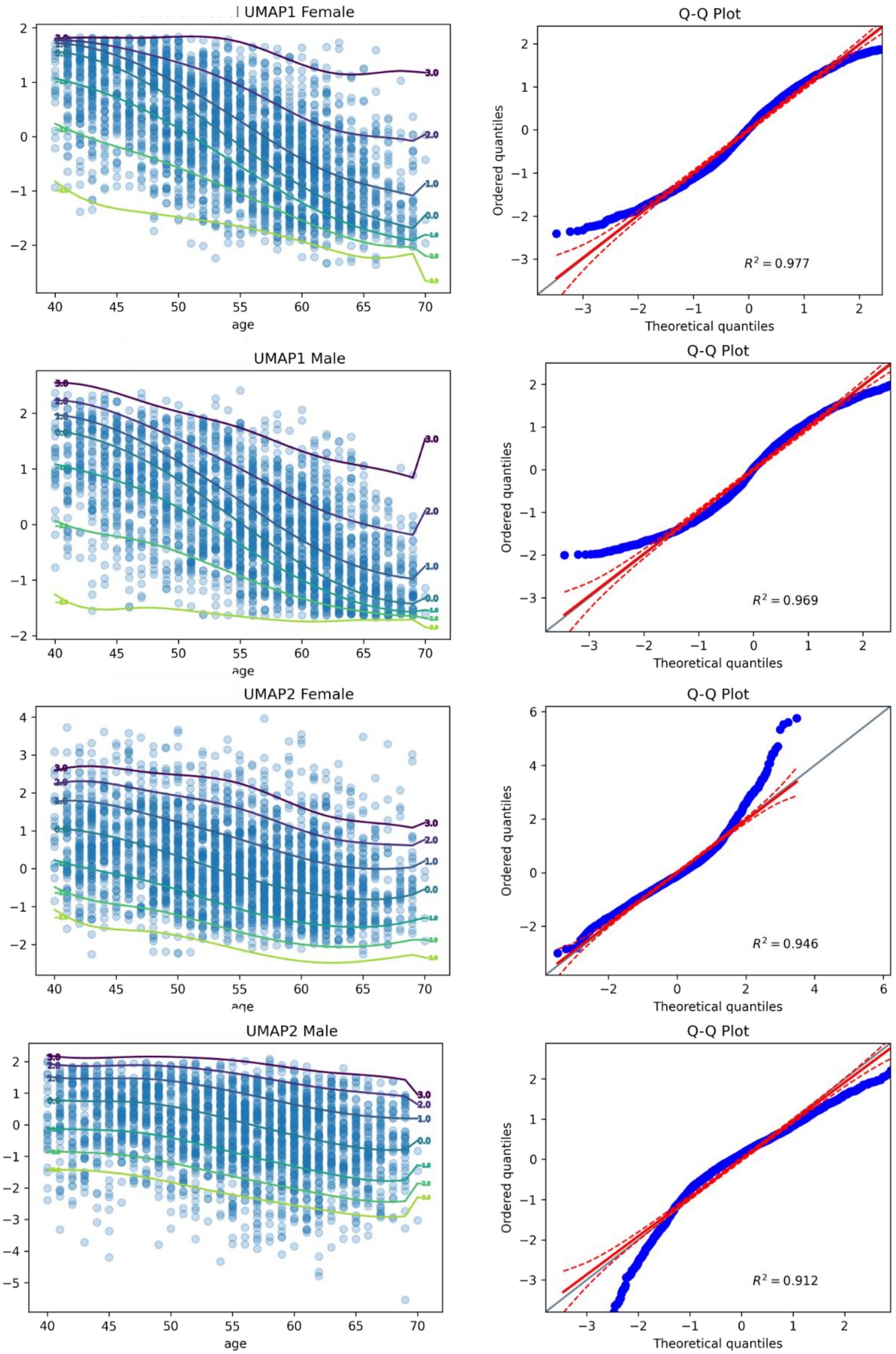

S7 Fig: normative UMPAs and qqplot of z-value of normative model

2-8 Association of the latent representation with non-imaging variables in UKB

S8 Fig shows the effect size of correlation of nIDPs and latent representation index for semi-supervised autoencoder and PCA.

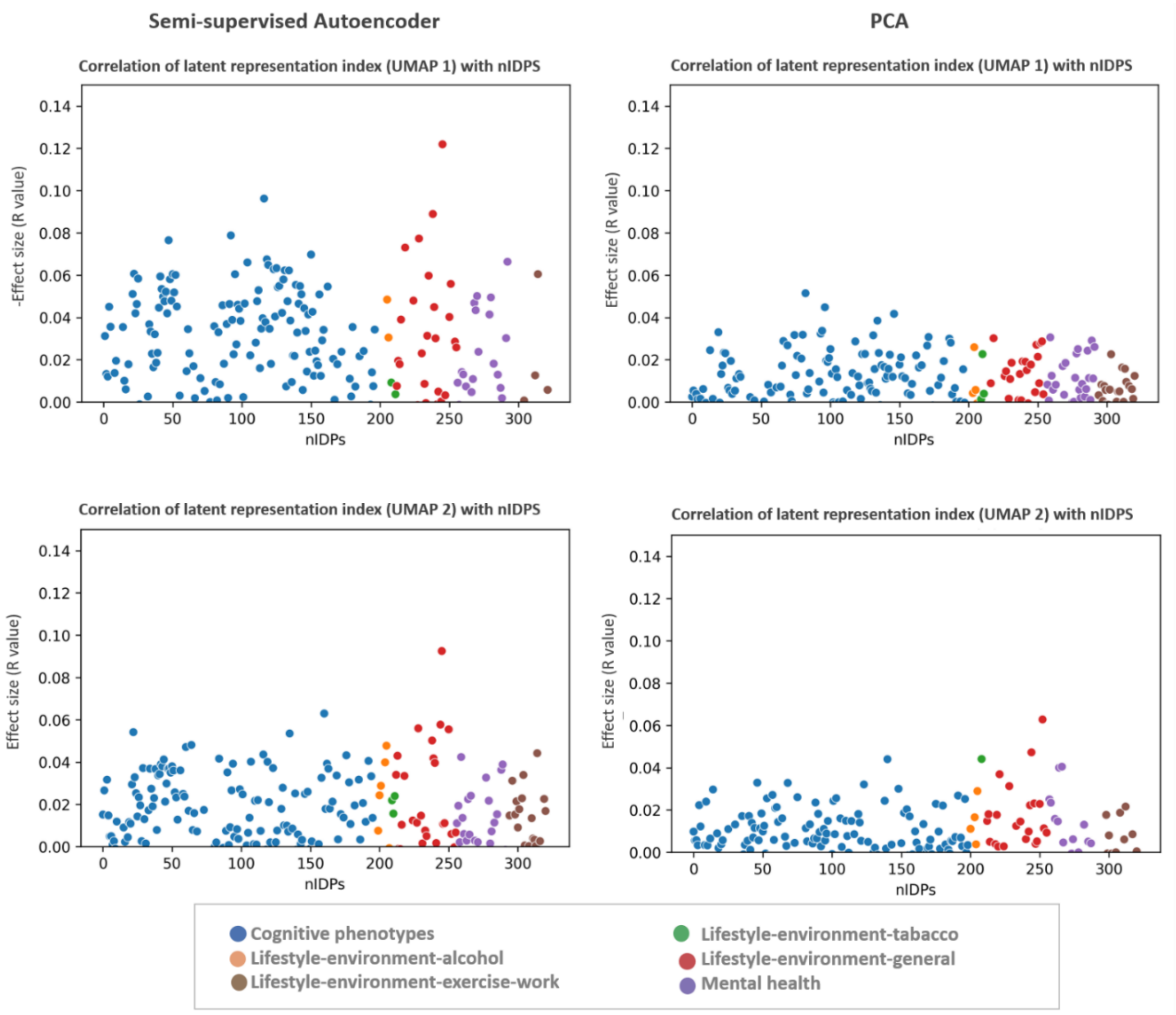

S8 Fig: Effect size of univariate correlation of non-imaging measures with the individualized deviations from normative UMAPs of latent space (latent representation index)

S9 Fig shows -10log(p_value) of correlation of latent representation of autoencoder with nIDPs. Here, there is highly level of association with age/sex information and other cognitive and behavioral measures. Hence, it shows the necessity of removing the confounding effect of age/sex from the latent variables. S10 and S11 Figs indicate that compared to standard PCA, the end results of mixed PCA hardly improved over standard PCA.

Moreover, similar analysis for ICAs and Sub-Cortical ROIs barely show significant associations.

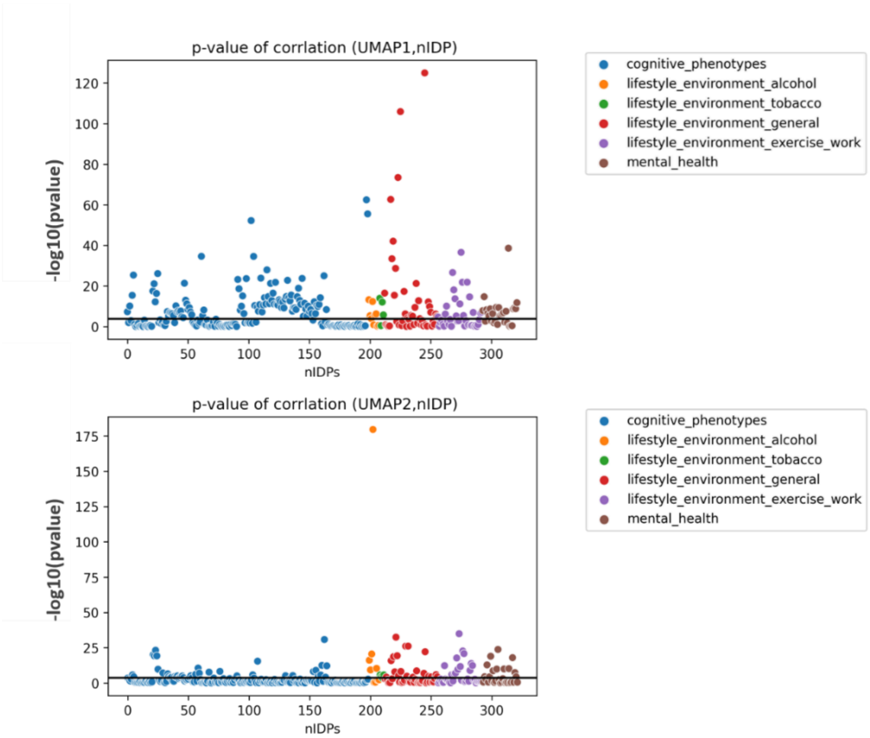

S9 Fig: Manhattan plot of -10log(p-value) of univariate correlation of non-imaging measures and UMAPs of latent representations of autoencoder. The black line is Bonferroni-corrected p-value threshold.

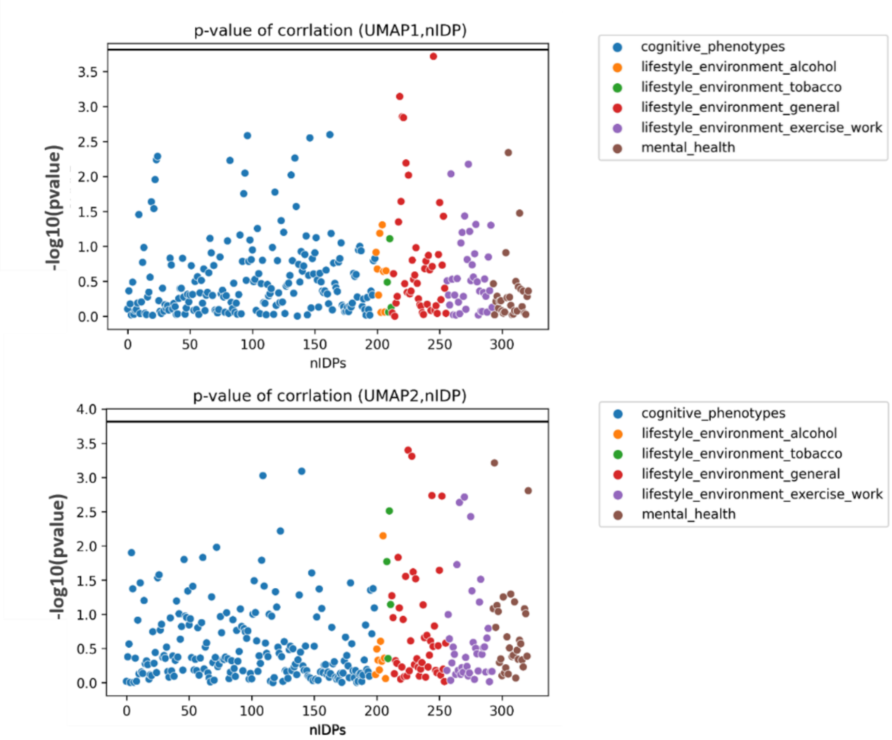

S10 Fig: Manhattan plot of -10log(p-value) of univariate correlation of non-imaging measures UMAPs of latent variables of vanilla-PCA with nIDPs . The black line is Bonferroni-corrected p-value threshold.

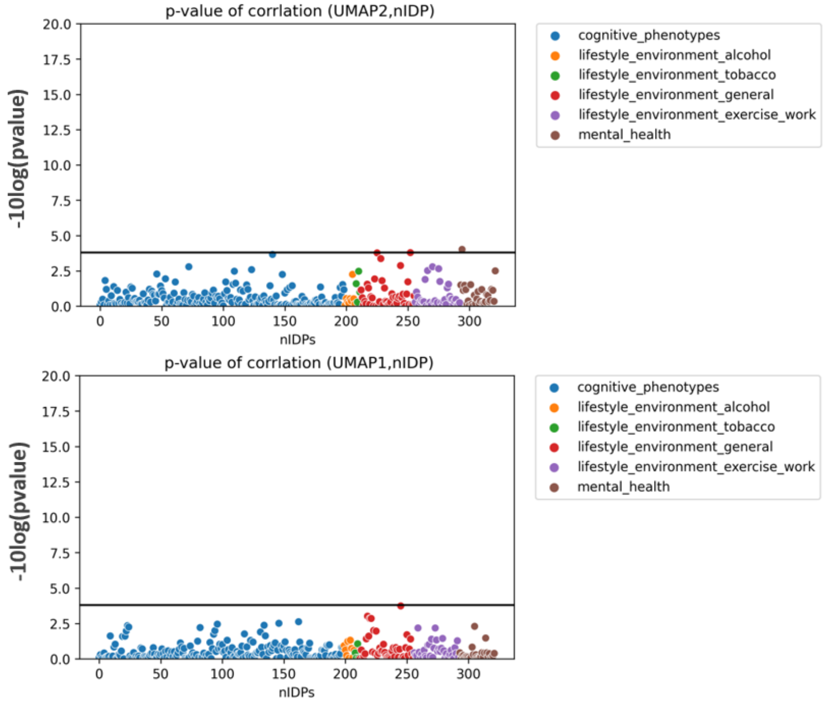

S11 Fig: Manhattan plot of -10log(p-value) of univariate correlation of non-imaging measures UMAPs of latent variables of mixed-PCA with nIDPs for 102(100). The black line is Bonferroni-corrected p-value threshold.

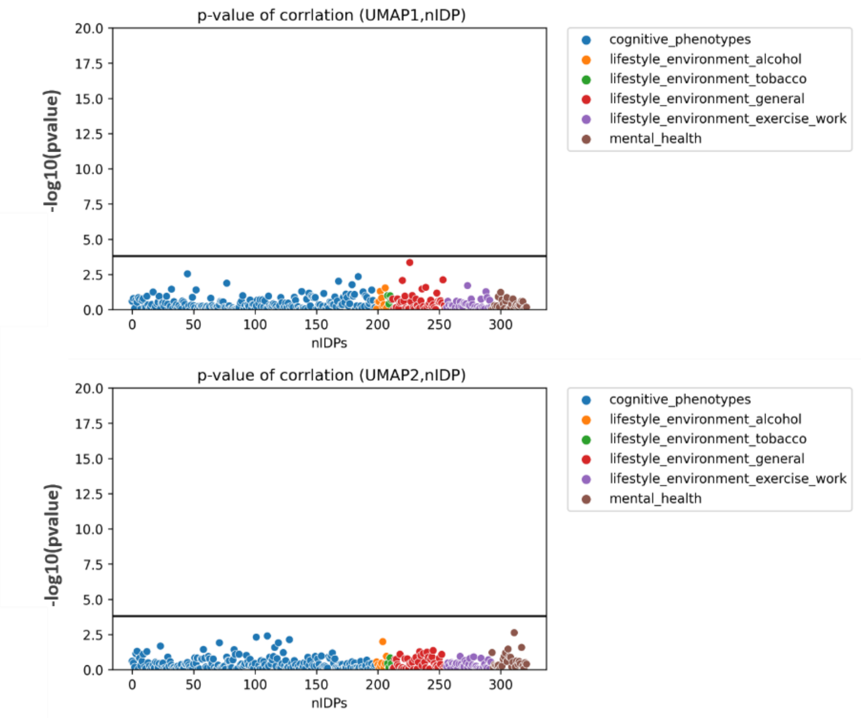

S12 Fig: Manhattan plot of -10log(p-value) of univariate correlation of non-imaging measures and UMAPs of ICAs. The black line is Bonferroni-corrected p-value threshold.

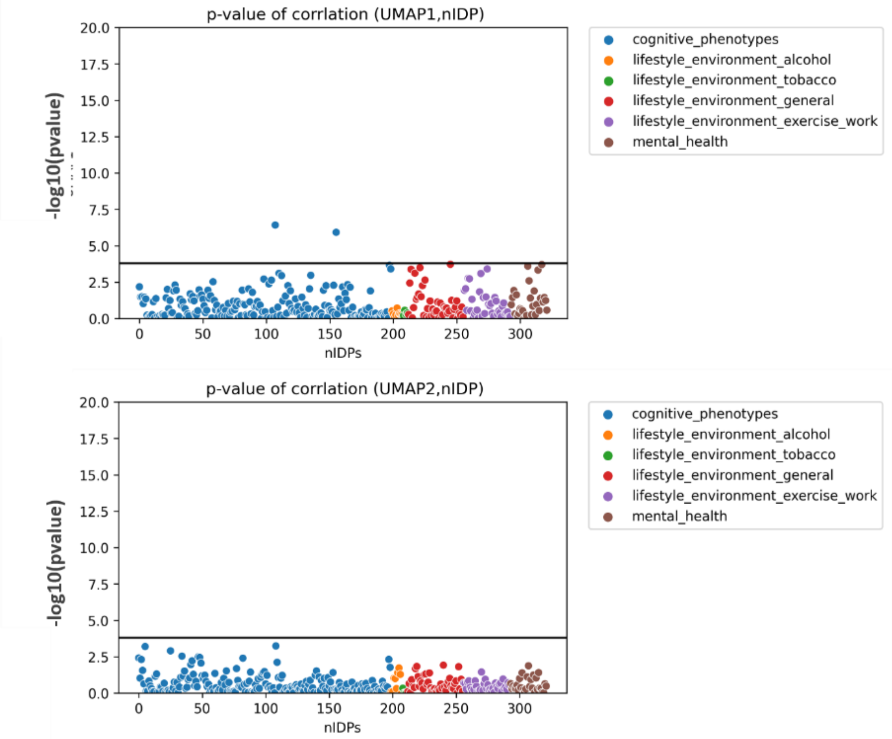

S13 Fig: Manhattan plot of -10log(p-value) of univariate correlation of non-imaging measures and UMAPs of sub-cortical ROIs. The black line is Bonferroni-corrected p-value threshold.

Supplementary References:

1. Barch DM, Burgess GC, Harms MP, Petersen SE, Schlaggar BL, Corbetta M, et al. Function in the human connectome: Task-fMRI and individual differences in behavior. Neuroimage. 2013;80:169–89.
2. Miller KL, Alfaro-Almagro F, Bangerter NK, Thomas DL, Yacoub E, Xu J, et al. Multimodal population brain imaging in the UK Biobank prospective epidemiological study. Nat Neurosci. 2016;19(11):1523–36.
3. Zabihi M, Oldehinkel M, Wolfers T, Frouin V, Goyard D, Loth E, et al. Dissecting the Heterogeneous Cortical Anatomy of Autism Spectrum Disorder Using Normative Models. Biol Psychiatry Cogn Neurosci Neuroimaging [Internet]. 2019 Jun;4(6):567–78. Available from: https://linkinghub.elsevier.com/retrieve/pii/S245190221830329X
4. Jones MC, Pewsey A. Sinh-arcsinh distributions. Biometrika. 2009;96(4).
5. Boer AAA de, Kia SM, Rutherford S, Zabihi M, Fraza C, Barkema P, et al. Non-Gaussian Normative Modelling With Hierarchical Bayesian Regression. bioRxiv. 2022;
6. Fraza CJ, Dinga R, Beckmann CF, Marquand AF. Warped Bayesian linear regression for normative modelling of big data. Neuroimage [Internet]. 2021 Dec 15 [cited 2022 Jun 12];245. Available from: https://pubmed.ncbi.nlm.nih.gov/34798518/
